# Tetrahalidometallate(II) Ionic Liquids with More than One Metal: The Effect of Bromide versus Chloride

**DOI:** 10.1002/chem.202201068

**Published:** 2022-09-15

**Authors:** Christian Balischewski, Biswajit Bhattacharyya, Eric Sperlich, Christina Günter, Alkit Beqiraj, Tillmann Klamroth, Karsten Behrens, Stefan Mies, Alexandra Kelling, Susanne Lubahn, Lea Holtzheimer, Anne Nitschke, Andreas Taubert

**Affiliations:** ^1^ Institute of Chemistry University of Potsdam 14476 Potsdam Germany; ^2^ Institute of Geosciences University of Potsdam 14476 Potsdam Germany

**Keywords:** electrochemistry, ionic liquids, metal-containing ionic liquids, *N*-butylpyridinium bromide, tetrahalidometallates

## Abstract

Fifteen *N*‐butylpyridinium salts – five monometallic [C_4_Py]_2_[MBr_4_] and ten bimetallic [C_4_Py]_2_[M_0.5_^a^M_0.5_^b^Br_4_] (M=Co, Cu, Mn, Ni, Zn) – were synthesized, and their structures and thermal and electrochemical properties were studied. All the compounds are ionic liquids (ILs) with melting points between 64 and 101 °C. Powder and single‐crystal X‐ray diffraction show that all ILs are isostructural. The electrochemical stability windows of the ILs are between 2 and 3 V. The conductivities at room temperature are between 10^−5^ and 10^−6^ S cm^−1^. At elevated temperatures, the conductivities reach up to 10^−4^ S cm^−1^ at 70 °C. The structures and properties of the current bromide‐based ILs were also compared with those of previous examples using chloride ligands, which illustrated differences and similarities between the two groups of ILs.

## Graphical abstract

**A question of halide**: Transition metal‐based ionic liquids (ILs) are promising candidates for electrochemical energy. Bromide‐based ILs are particularly interesting for their high electrochemical stability, although their conductivities are significantly lower than those of their chloride analogues. [MBr_4_]^2−^ units are therefore highly promising building blocks for advanced electrochemically active ILs.

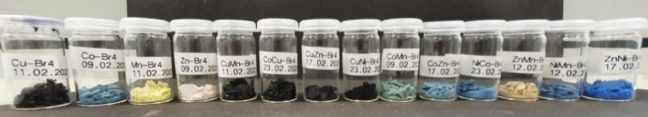

## Introduction

Ionic liquids (ILs) have gathered a lot of interest; this is due to their interesting and sometimes surprising properties. ILs are salts with rather low melting points below about 100 °C. This is mainly due to weaker interactions between the constituting ions compared to conventional salts. The weakening of the interactions originates from steric effects mostly caused by the cations and charge delocalization in both anions and cations. As a consequence, ILs have much lower melting points than most conventional (inorganic) salts. ILs are effective solvents for organic and inorganic compounds, display high ionic conductivity, often good electronic conductivity, and often have a broad electrochemical stability window.[^1^, ^2^, ^3^, ^4^] Due to their low flammability and low vapor pressure, ILs are often safe and easy to handle.[^4^, ^5^] Moreover, ILs have gained popularity since their chemical, physical or biological properties can easily be adjusted by changing the anion or cation, or by altering and/or adding specific functional groups.[^2^, ^5^] As a result, ILs are often used as electrolytes in batteries, fuel cells, actuators and photovoltaic cells, or used as solvents or catalysts thus demonstrating a very broad application potential.[^6^, ^7^, ^8^, ^9^]

Metal‐containing ILs (MILs), that is, ILs including at least one metal ion in the anion or cation, have attracted growing attention in the recent past.^[10]^ Due to the presence of metals, MILs possess interesting and highly variable catalytic, electrochemical, magnetic, and optical properties, with some of them being unique to MILs compared to other ILs.[^3^, ^5^, ^11^, ^12^, ^13^, ^14^, ^15^, ^16^, ^17^, ^18^, ^19^, ^20^, ^21^] While there are already numerous studies on main group metal ILs,^[22]^ transition metal‐based MILs show increasing interest for the above applications and beyond. This is mostly due to the fact that the transition metals provide access to a wide variety of properties that can be imparted to the ILs through, for example, the diverse redox properties of the transition metal constituents. Moreover, MILs are not just interesting materials in their own right, they can also act as starting materials for the synthesis of a large variety of inorganic or hybrid materials like carbon/inorganic composites or metal sulfides.[^19^, ^23^, ^24^, ^25^, ^26^, ^27^] MILs therefore are very useful and highly adaptable ionic liquid precursors (ILPs) for the synthesis of a large variety of (nano)materials such as sulfides, selenides, oxides, or perovskite structures for, for example, use in green energy conversion.[^28^, ^29^]

The [MX_4_]^*n*−^ unit (M=Cu, Co, Mn, Ni, Zn, Pd, In, Ga, Cd, Al, Fe, etc.; X=Cl, Br, I; *n*=1, 2, 3) is the most common building block for the anion in MILs. Those halidometallate ILs present arguably the largest subgroup of MILs studied, with chloridometallate ILs being the most common ones.[^10^, ^17^, ^30^, ^31^, ^32^, ^33^, ^34^, ^35^, ^36^, ^37^, ^38^, ^39^]

Although MILs have attracted a lot of attention, there are only few studies on the influence of metals, metal combinations, or type of halide on optical, electrochemical, thermal, and structural properties. Abouserie et al.^[10]^ studied the influence of three different transition metals in monometallic ILs on thermal and structural properties. Kore et al.^[22]^ made bimetallic ILs using transition metals and group III metals. Recently, Liu et al.^[40]^ reported mixed MILs by adding a metal salt to a monometallic MIL, that is, an IL based on only one metal ion type. Moreover, Yoshida et al.^[41]^ studied the influence of halides on the physical properties of paramagnetic imidazolium‐based MILs. None of these studies, however, analyzes the influence of multiple metals or different halides on the thermal, structural, optical, and especially electrochemical properties of the ILs at room and elevated temperatures.

As a result, there is basically no information on the correlation between chemical composition, details of the resulting structure, and the optical, electronic or magnetic properties of the MILs. We have recently reported the first data of chloride‐based MILs with a series of d‐metals and their mixtures and demonstrated a strong influence of the metal cation on the properties of the MILs.^[42]^ In line with previous data, the *N*‐butylpyridinium cation was used as the counterpart of the tetrabromidometallate (II) anion. This allows for comparisons with the chloride analogues published before and ensures a higher stability and higher melting points of the compounds compared to imidazolium‐based compounds.[^43^, ^44^, ^45^] Moreover, previous studies showed that the use of bigger cations compared to pyridinium may not result in ILs.[^44^, ^45^] Rather, they result in crystalline compounds which decompose at elevated temperatures. The current article focuses on the structural, thermal and electrochemical properties of much less well established bromidometallate(II) MILs and further expands the pool of these interesting and useful compounds.

## Results and Discussion

**Figure 1:**
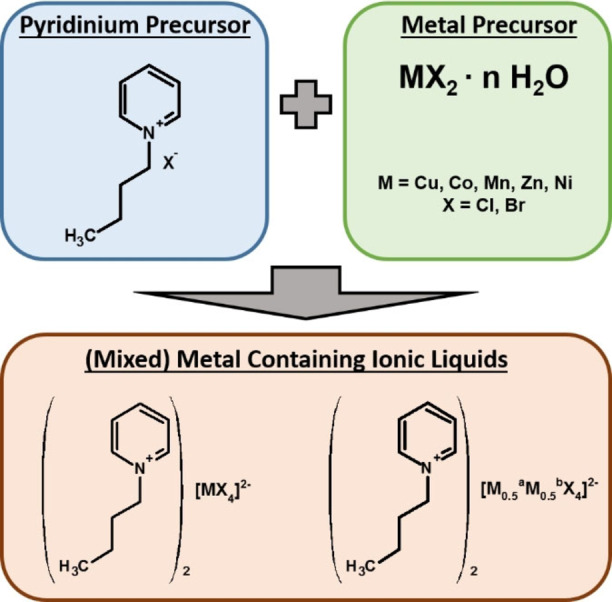
Synthesis protocol for the MILs with M^a^ and M^b^ being different metals, as listed below.

All MILs were synthesized according to established protocols (Figure 1). At room temperature, all MILs are solid with melting points ranging from 64 to 101 °C.

Inductively coupled plasma optical emission spectrometry (ICP‐OES) data, Table 1, show that the experimentally obtained compositions almost exactly match the calculated compositions with a general mismatch between calculated and experimental metal content of under 2 atom%. **IL10** (theoretical Zn/Ni composition of 50 : 50) is the single exception showing a higher deviation of roughly 8 atom%. This correlates well with previous data^[42]^ showing that the synthetic approach is clearly capable and viable for the synthesis of MILs containing multiple metals in a defined ratio.

**Table 1:** Metal composition of the MILs determined from ICP‐OES measurements.

Compound^[a]^	Abbreviation	Cu [atom%]	Co [atom%]	Mn [atom%]	Ni [atom%]	Zn [atom%]
(C_4_Py)_2_[Cu_0.5_Mn_0.5_Br_4_]	**IL1**	50.75±0.78	–	49.25±0.78	–	–
(C_4_Py)_2_[Cu_0.5_Co_0.5_Br_4_]	**IL2**	50.03±0.40	49.97±0.40	–	–	–
(C_4_Py)_2_[Cu_0.5_Zn_0.5_Br_4_]	**IL3**	49.78±0.40	–	–	–	50.22±0.40
(C_4_Py)_2_[Cu_0.5_Ni_0.5_Br_4_]	**IL4**	48.24±0.09	–	–	51.76±0.09	–
(C_4_Py)_2_[Co_0.5_Mn_0.5_Br_4_]	**IL5**	–	50.51±0.41	49.49±0.41	–	–
(C_4_Py)_2_[Co_0.5_Zn_0.5_Br_4_]	**IL6**	–	50.09±0.52	–	–	49.91±0.52
(C_4_Py)_2_[Co_0.5_Ni_0.5_Br_4_]	**IL7**	–	48.89±0.49	–	51.11±0.49	–
(C_4_Py)_2_[Mn_0.5_Ni_0.5_Br_4_]	**IL8**	–	–	49.38±0.07	50.62±0.07	–
(C_4_Py)_2_[Mn_0.5_Zn_0.5_Br_4_]	**IL9**	–	–	49.91±0.52	–	50.09±0.52
(C_4_Py)_2_[Zn_0.5_Ni_0.5_Br_4_]	**IL10**	–	–	–	42.70±0.20	57.30±0.20
(C_4_Py)_2_[CuBr_4_]^[b]^	**IL11**	–	–	–	–	–
(C_4_Py)_2_[CoBr_4_]^[b]^	**IL12**	–	–	–	–	–
(C_4_Py)_2_[MnBr_4_]^[b]^	**IL13**	–	–	–	–	–
(C_4_Py)_2_[NiBr_4_]^[b]^	**IL14**	–	–	–	–	–
(C_4_Py)_2_[ZnBr_4_]^[b]^	**IL15**	–	–	–	–	–

[a] Theoretical composition from synthesis. [b] No ICP‐OES measurements were made for the monometallic ILs as no other metals can be expected in these compounds. They are listed for completeness and for identifying the respective IL labels. Note: **IL13a** describes the chloride analogue of **IL13** (see Figure 5, below).

**Figure 2:**
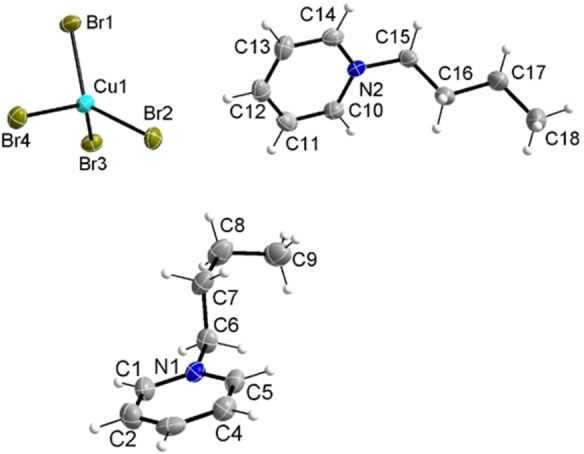
Molecular structure of (C_4_Py)_2_[CuBr_4_] with atomic labels. Displacement ellipsoids are shown at the 50 % probability level.

Single‐crystal X‐ray diffraction shows that **IL11** crystallizes in the monoclinic space group *P*2_1_/*n* with four formula units per unit cell, identical to previous examples such as (C_4_Py)_2_[CuCl_4_].^[42]^ However, the cell parameters and the arrangement of the ions differ in both compounds. Table 2 summarizes the crystallographic and refinement data of **IL11**. The asymmetric unit is shown in Figure 2.

**Table 2:** Crystal data and details of structure refinement for (C_4_Py)_2_[CuBr_4_] (**IL11**).

	(C_4_Py)_2_[CuBr_4_]
empirical formula	C_18_H_28_Br_4_CuN_2_
*M* [g mol^−1^]	655.60
*T* [K]	210
*λ* [Å]	0.71073 (Mo_Kα_)
crystal system	monoclinic
space group	*P*2_1_/*n*
unit cell dimensions	
*a* [Å]	9.4918(19)
*b* [Å]	18.706(4)
*c* [Å]	13.265(3)
*α* [°]	90
*β* [°]	94.96(3)
*γ* [°]	90
*V* [Å^3^]	2346.4(8)
*Z*	4
*ρ*_calc_ [g cm^−3^]	1.856
*μ* [mm^−1^]	7.76
*F*(000)	1276
crystal description	black, block
crystal size [mm^3^]	0.30×0.23×0.10
*θ*_min_/*θ*_max_ [°]	3.0/25.0
Index ranges	−11≤*h*≤11
	−22≤*k*≤21
	−15≤*l*≤14
reflection collected	39862
independent reflection	4127
*R*_int_	0.077
reflections *I*>2*σ*(*I*)	2827
parameter	231
*R*_1_/*wR*_2_ [*I*>2*σ*(*I*)]	0.0318/0.0552
*R*_1_/*wR*_2_ [all data]	0.0673/0.0637
min./max. Δ*ρ*	
[10^−6^ e pm^−3^]	−0.42/0.41
GooF	1.000
CCDC	2152368

**Figure 3:**
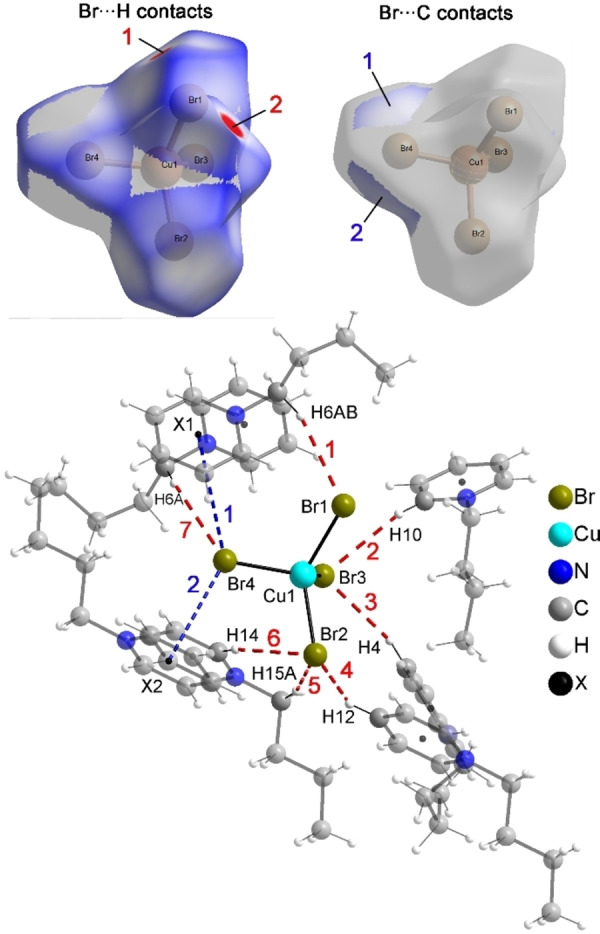
Hirshfeld surface of the [CuBr_4_]^2−^ anion for the Br⋅⋅⋅H contacts (top, left) and the Br⋅⋅⋅C contacts (top, right) and intermolecular interactions between the anion and the cations (below) with hydrogen bonds (red) and anion–π interactions (blue).

To determine the short intermolecular contacts between the ions in **IL11**, the Hirshfeld surface of the anion was analyzed.^[46]^ Figure 3 (top) shows the Hirshfeld surface area of the [CuBr_4_]^2−^ anion for the Br⋅⋅⋅H contacts (left) and the Br⋅⋅⋅C contacts (right) with a *d*_norm_ range of −0.07 to 1.0 Å.

The arrangement of the cations and anions in the solid state occurs with the formation of C−H ⋅ ⋅ ⋅ Br hydrogen bonds and anion–π interactions between the bromide ligands of the anions and the aromatic ring of the cations. Figure 3 (bottom) shows the environment of the symmetry‐independent anion with hydrogen bonds visualized in red dashed lines, anion–π interactions in blue dashed lines and the center of the aromatic ring shown as black dot and labeled as X. The bond distances and angles of the interactions are summarized in Table 3. With H⋅⋅⋅Br distances ranging from 2.81 to 3.00 Å and C−H⋅⋅⋅Br angle far below 180° the hydrogen bonds are weak, even weaker than those observed in similar compounds.^[47]^ The Br atom labeled Br4 forms two anion–π interactions to the aromatic rings of two neighboring butyl‐pyridinium cations with Br⋅⋅⋅X distances of 4.083 and 3.672 Å.^[48]^ These short distances and the resulting N⋅⋅⋅X⋅⋅⋅Br angles of nearly 90° suggest, that the interactions are relative strong and may be preferred over weak C−H⋅⋅⋅Br hydrogen bonds.

**Table 3:** Distances and angles of the intermolecular interactions in (C_4_Py)_2_[CuBr_4_] (**IL11**).

Hydrogen bonds	D−H [Å]	H⋅⋅⋅A [Å]	D⋅⋅⋅A [Å]	D−H⋅⋅⋅A [°]
1 (C6−H6AB⋅⋅⋅Br1)	0.98	2.86	3.795 (4)	161
2 (C10−H10⋅⋅⋅Br3)	0.94	2.81	3.614 (4)	144
3 (C4−H4⋅⋅⋅Br3)	0.94	2.96	3.727 (4)	140
4 (C12−H12⋅⋅⋅Br2)	0.94	3.00	3.604 (4)	124
5 (C15−H15 A⋅⋅⋅Br2)	0.98	2.98	3.930 (4)	164
6 (C14−H14⋅⋅⋅Br2)	0.94	2.95	3.845 (4)	160
6 (C6−H6 A⋅⋅⋅Br4)	0.98	2.99	3.874 (4)	151
Anion–π interactions	Br⋅⋅⋅X [Å]			N⋅⋅⋅X⋅⋅⋅Br [°]
1 (Br4⋅⋅⋅X1)	4.08			90.6
2 (Br4⋅⋅⋅X2)	3.67			90.9

**Figure 4:**
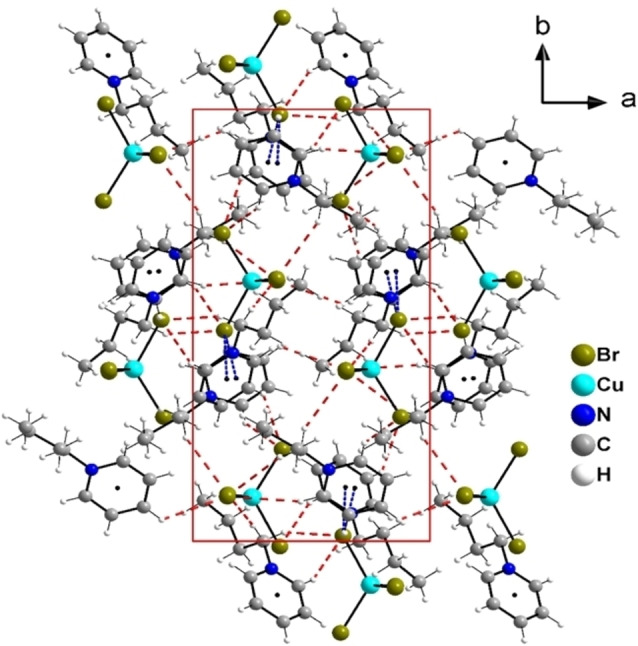
Representation of the unit cell of (C_4_Py)_2_[CuBr_4_] (**IL11**) viewed along the crystallographic *c*‐axis.

The unit cell of **IL11** is shown in Figure 4. Due to the absence of strong directional intermolecular interactions, the arrangement of the anions in the unit cell is relatively diffuse. In contrast to the chloride‐coordinated compound (C_4_Py)_2_[CuCl_4_],^42^ the arrangement of the ions does not lead to a formation of chains or sheets in (C_4_Py)_2_[CuBr_4_]. This observation agrees with the weak crystallinity (Figure S1 in the Supporting Information) and the low melting point of **IL11** of 64.7 °C which is slightly lower than the melting point of (C_4_Py)_2_[CuCl_4_] with 67.0 °C.

**Figure 5:**
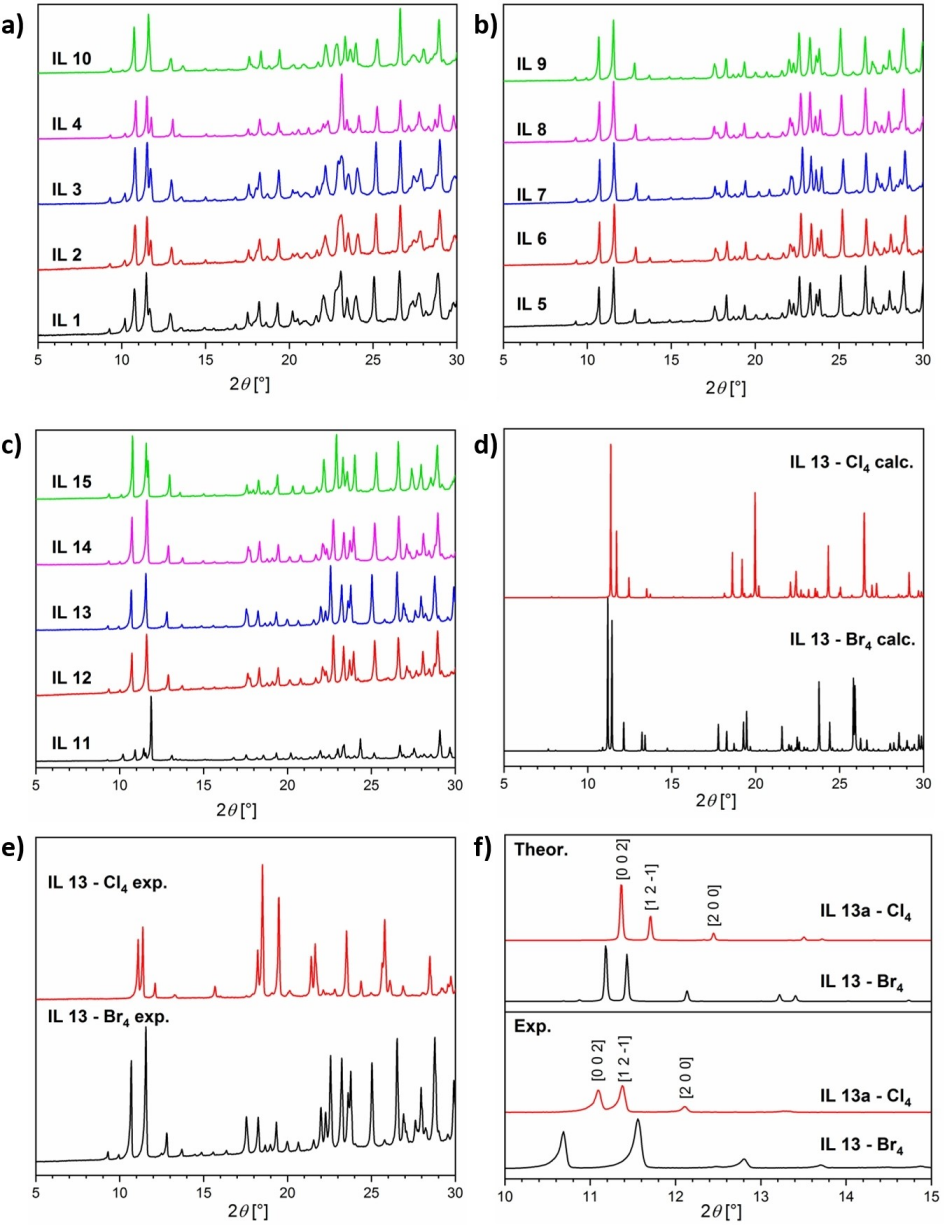
X‐ray diffractograms of a), b) the mixed MILs **1**–**10** (Table 1) and c) the monometallic MILs **11**–**15** (Table 1). d) A comparison between calculated manganese‐ILs containing either chloride or bromide, as calculated and optimized by using DFT. e) A comparison between a synthesized chloride‐containing and a bromide‐containing manganese‐IL (**IL13**, Table 1). f) A detailed comparison of the theoretical and experimental data on **IL13** in the region of 10–15° 2*θ* also showing the corresponding *hkl* values. XRD measurements for (e) and (f) were made by using repetitive measurements for a better signal‐to‐noise ratio and better resolution. The *y*‐axis shows the intensity (in a.u.). Note: The copper‐containing MILs (**IL1**–**4** and **IL11**) show reflection splitting; this indicates a possible distortion or deterioration of the monoclinic space group.

The crystal structure of all ILs was further analyzed using powder X‐ray diffraction (XRD), Figure 5. The XRD data show that all compounds are solid and crystalline at room temperature. Furthermore, all [MBr_4_]^2−^ based ILs are isostructural, Figure 5a–c. While the intensity of the reflections differs to some extent, which is probably due a high number of defects and disorder in each sample, the position of the reflections are identical. As with previously published data on chloride‐containing MILs,^[42]^ the background of the patterns is relatively noisy.

Moreover, similar to previous studies,[^10^, ^42^] the exchange of one 3d‐metal cation for another one (or for multiple cations) does not result in significant changes in the crystal structure. Interestingly, a comparison of the diffractograms of a Mn‐based MIL containing chloride with a Mn‐based MIL containing bromide (**IL13**; Figure 5e) shows minor differences in the reflection positions and intensities. The shifts indicate a change in the cell dimension. We currently attribute the shifting peak positions to the change of the bond length between copper and the two halides. The change in the reflection intensities can be attributed to a change in the bond angles and the inclusion of different atoms.

This hypothesis is supported by theoretical calculations of the two manganese ILs (**IL13**, Table 1) with one containing chloride and the other containing bromide, as shown in Figure 5d. Here, the unit cells were calculated using DFT with the PBE functional, by calculating the diffractograms with the entropy method from optimized unit cell structures. The calculations show that the diffractograms display a high degree of agreement. The differences to the measured diffractograms (Figure 5e) are due to the calculated diffractograms being optimized at 0 K and 0 atm.

All MILs crystallize in the same space group *P*2_1_/*n*. The chloride‐containing ILs[^10^, ^42^] and the bromide‐containing ILs (Table 1) can be classified as being isostructural within their groups, but this does not fully apply to the comparison of both MIL groupings. However, there is a high degree of agreement and conformity between the MILs containing chloride or bromide in terms of their structure. Moreover, XRD shows that traces of *N*‐butylpyridinium bromide remain in the powders (Figure S2).

Furthermore, a more detailed comparison of the theoretical and experimental XRD data between 10 to 15° 2*θ* obtained for **IL13** is displayed in Figure 5f. There is a good agreement between the experimental and calculated diffractograms using theoretical structures obtained by DFT. Comparing the reflection positions of three prevalent lattice planes (cell parameters in Table S2), Tables 4 and S1, it is clear that the shifts in the patterns between chloride and bromide‐based compounds is minimal with the highest being 0.6° 2*θ* for the experimental data and 0.3° 2*θ* for the theoretical data.

**Table 4:** Comparison of reflection position and *d* values of the corresponding *hkl* values from theoretical and experimental data on **IL13**.

Structure	**[*h k l*]**		
	2*θ* [0 0 2]	2*θ* [1 2 −1]	2*θ* [2 0 0]
**IL13**–Br_4_ calc.	11.18	11.43	12.14
**IL13**–Cl_4_ calc.	11.36	11.71	12.45
Δ_2*θ*_	0.18	0.28	0.31
			
**IL13**–Br_4_ exp.	10.69	11.56	12.81
**IL13**–Cl_4_ exp.	11.09	12.16	13.30
Δ_2*θ*_	0.40	0.60	0.49
Structure	**[*h k l*]**		
	*d* [0 0 2] in Å	*d* [1 2 −1] in Å	*d* [2 0 0] in Å
**IL13**–Br_4_ calc.	7.9	7.7	7.3
**IL13**–Cl_4_ calc.	7.8	7.6	7.1
Δ_d_	0.1	0.1	0.2

This is expected due to the different ionic radii of chloride (181 pm) and bromide (196 pm), which impact the unit cell volume and thus the cell parameters. The deviations between theoretical and experimental data are also expected due the different conditions (0 K, 0 atm) of DFT. Consequently, the comparison of the theoretical and experimental data shows that the theoretical approach is highly viable and produces promising data. This is especially valuable when working with MILs, which typically do not crystallize easily and it is therefore not always possible to obtain good quality crystals for X‐ray analysis.

Additionally, it is important to keep in mind that the starting point for the DFT calculations was the previously published copper‐chloride‐containing MIL, which possesses a different albeit isostructural and isoelectronic anion.^42^ This also shows the transferability of the method from better‐known compounds to lesser known structures.

**Figure 6:**
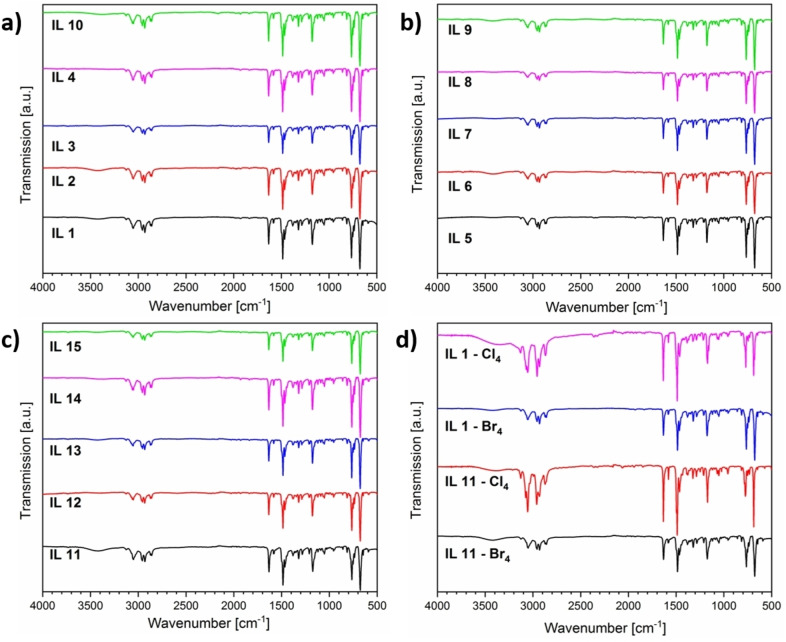
Infrared spectra of a), b) the mixed MILs **1**–**10** (Table 1) and c) the monometallic MILs **11**–**15** (Table 1). d) A comparison between a mixed (**IL1**‐Cl_4_) and a monometallic IL (**IL11**‐Cl_4_) containing chloride and a mixed (**IL1**‐Br_4_) and a monometallic IL (**IL11**‐Br_4_) containing bromide. The *y*‐axis shows the transmission (in a.u.).

Figure 6a–c shows the IR spectra of the MILs. Similar to the chloride‐containing MILs,^[42]^ the spectra of the individual MILs are identical. Essentially the only difference can be found between 3500 and 2800 cm^−1^ with varying intensities of the absorption bands. These signals can be attributed to the presence of water in the MILs, which is not surprising, as ILs and MILs are known for their hygroscopicity.[^42^, ^49^]

Furthermore, all MILs show the typical absorption bands at 1630, 1580, and 1490 cm^−1^ that can be assigned to the aromatic C=C and C=N stretching vibrations of the pyridinium ring. The typical O−H deformation vibrations between 1390 and 1210 cm^−1^ further indicate the presence of water. Bands at 1170 and 958 cm^−1^ are typical for (exocyclic) C−N stretching vibrations of alkylated pyridinium salts. Additionally, aromatic deformation vibrations can be observed at 770, 730 and 680 cm^−1^. Overall, IR spectroscopy confirms the presence of the pyridinium cations in the compounds and also strongly indicates the presence of water within the solids.

In addition, Figure 6d further compares the IR spectra of existing chloride‐containing MILs^[42]^ and the newer bromide‐containing MILs using a pure copper MIL and a mixed copper‐manganese MIL (**IL1** and **IL11**, Table 1) as an example. Clearly, the IR spectra perfectly match with the only difference being the amount of water in the samples as shown by the intensity of the absorption bands between 3500 and 2800 cm^−1^. Thus, the properties and behavior of the organic cation in the bromide‐containing MILs is identical to the organic part of the chloride‐containing MILs. This illustrates that the [MX]_4_^2−^ anion clearly is the building block responsible for the properties of each IL.

**Figure 7:**
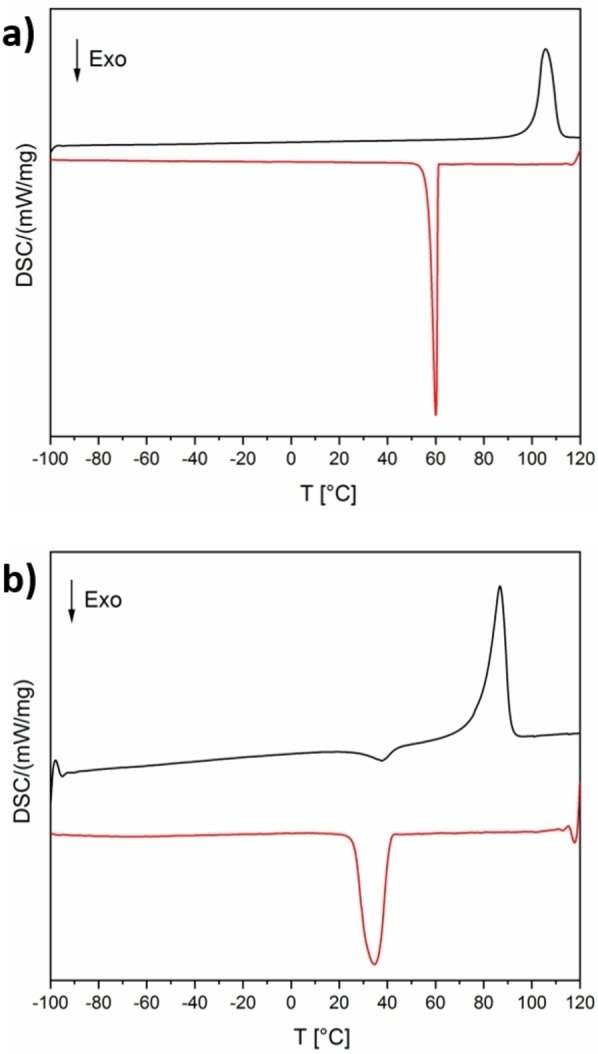
Representative DSC data (second heating (black) and cooling (red) run) of a) **IL13** and b) **IL4**.

Besides the IL structure, thermal properties were analyzed as well. Figure 7a and b shows representative differential scanning calorimetry (DSC) data, with the remaining being summarized in Table S3. All MILs have melting points between 64 and 101 °C, which is in line with previous data.[^10^, ^42^] It has to be noted, however, that some of the Cu‐ and Ni‐containing MILs show a rather broad melting peak with a minor part of the melting process occurring at lower temperatures between 50 and 70 °C. As in other cases, this can be assigned to smaller crystallites melting at lower temperatures (pre‐melting). Furthermore, copper‐containing MILs (**IL1**–**4** and **IL11**) show a cold crystallization during the heating cycle.

Moreover, all MILs show lower melting points than pure *N*‐butylpyridinium chloride (*T*_M_=160 °C) and pure *N*‐butylpyridinium bromide (*T*_M_=108 °C). The compounds containing copper occasionally show glass transition points and cold crystallization processes, which are well known for ILs.^[50]^ The copper‐containing MILs also show the lowest melting points, which may be the result of the Cu^II^ d^9^ system exhibiting Jahn–Teller distortions.[^10^, ^44^, ^45^, ^51^] As a result of Jahn–Teller distortions, systems like Cu^II^ d^9^ show a tendency of an s–d promotion. This leads to an increase of so‐called “preferred liquid structures” that possess partially filled d‐bands and act as impurities, which leads to lower free energies and thus, for example, lower melting temperatures.[^52^, ^53^, ^54^] After repeated heating and cooling, the copper‐containing MILs show a broadening in the crystallization peak combined with the delayed crystallization during the heating cycle. Interestingly, manganese seems to prevent or hamper the delayed crystallization in copper‐containing samples. Upon cooling, the crystallization temperature can be observed between −1 and +54 °C, depending on the sample.

Additionally, thermogravimetric analysis (TGA) data show that the MILs are thermally stable up to 200–250 °C, Figure S3. This is consistent with previous data on chloride‐based MILs.[^10^, ^42^, ^55^] The first weight loss is observed between 200 and 250 °C with a weight loss of around 55 % in **IL11** and 65 % in **IL13**. The second step occurs at 350 °C, however, only in **IL11**. Overall, a weight loss of around 70 to 90 % is observed for all MILs is observed.

At last, the electrochemical properties of the MILs were analyzed. Firstly, the electrochemical stability window (ESW) was determined using cyclic voltammetry (CV). The ESW is determined by analyzing the range within which the MILs show no irreversible changes from electrolysis or other decomposition. Furthermore, CV provides insights into oxidation and reduction processes between −1 to 3 V. All MILs were studied using a ferrocene reference standard in a dry acetonitrile solution to prevent redox processes of excess water.^[56]^ The potential limits were chosen to provide experimental parameters such that the data can be compared to previous data.^[42]^ Furthermore, the MILs show a significantly higher current above +3 V and notable lower values below −1 V, which might indicate decomposition processes above or below those limits.

**Figure 8:**
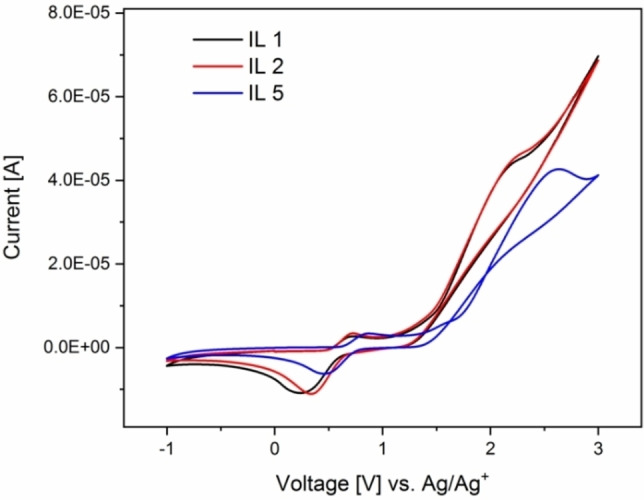
Cyclic voltammograms of **IL1**, **IL2** and **IL5** in acetonitrile with a ferrocene reference.

Interestingly, all bromide‐containing MILs studied here show a nearly identical redox behavior, Figure 8 with the only changes being slightly differing peak locations. All CVs show a reduction peak around 0.75±0.1 V, and an oxidation peak at 0.35±0.1 V. These signals can be assigned to the reduction/oxidation of the ferrocene reference.^[42]^ Besides the ferrocene signal, there is no other process that can be observed in this measurement. This indicates that the MILs are rather stable under these conditions. To further prove that the respective signals in the CV data are solely from the ferrocene, CVs without ferrocene were obtained as well, Figure S7. Here, no redox processes are visible; this indeed demonstrates that the only source of this particular signal is the ferrocene standard.

Secondly, the conductivities of the ILs were determined using temperature‐dependent impedance spectroscopy.[^57^, ^58^, ^59^, ^60^, ^61^] Here, the resistance values of the MILs used as electrolytes between two electrodes are measured and converted into the corresponding conductivities. Table 5 shows the impedance data as well as measurement parameters and the resulting conductivity values. The measurements of **IL11** and partly **IL1** did not result in analyzable data due to redox‐ and degradation processes during measurements. This observation correlates well with the behavior of the corresponding chloride‐containing copper‐IL.^[42]^

**Table 5:** Resistances and conductivities of the ILs obtained from impedance spectroscopy.

MIL	*T* [°C]^[a]^	*R* [Ω]^[b]^	*ΔR* [Ω]^[c]^	*σ* [S cm^−1^]^[d]^	MIL	*T* [°C]^[a]^	*R* [Ω]^[b]^	*ΔR* [Ω]^[c]^	*σ* [S cm^−1^]^[d]^
IL1	30	8.32×10^5^	±4.92×10^3^	1.18×10^−5^	IL9	30	3.03×10^6^	±3.68×10^4^	3.74×10^−6^
	40	2.92×10^5^	±5.84×10^2^	3.37×10^−5^		40	1.93×10^6^	±2.63×10^4^	5.87×10^−6^
	50	1.19×10^5^	±5.49×10^3^	8.29×10^−5^		50	1.27×10^6^	±1.86×10^4^	8.93×10^−6^
	60	–	–	–		60	8.11×10^5^	±1.29×10^4^	1.40×10^−5^
	70	–	–	–		70	1.88×10^5^	±1.09×10^3^	6.00×10^−5^
IL2	30	1.71×10^6^	±1.68×10^4^	6.29×10^−6^	IL10	30	1.50×10^6^	±5.60×10^3^	6.55×10^−6^
	40	6.97×10^5^	±5.14×10^3^	1.54×10^−5^		40	6.31×10^5^	±2.68×10^3^	1.56×10^−5^
	50	2.49×10^5^	±1.23×10^3^	4.32×10^−5^		50	2.95×10^5^	±1.06×10^3^	3.34×10^−5^
	60	5.70×10^4^	±8.60×10^1^	1.89×10^−4^		60	1.32×10^5^	±2.47×10^2^	7.43×10^−5^
	70	1.16×10^4^	±9.78	9.31×10^−4^		70	5.75×10^4^	±8.10×10^1^	1.71×10^−4^
IL3	30	1.07×10^7^	±3.87×10^4^	1.05×10^−6^	IL11	30	–	–	–
	40	3.71×10^6^	±1.31×10^4^	3.05×10^−6^		40	–	–	–
	50	1.05×10^6^	±3.68×10^3^	1.08×10^−5^		50	–	–	–
	60	1.91×10^5^	±3.25×10^2^	5.92×10^−5^		60	–	–	–
	70	1.76×10^4^	±1.72×10^1^	6.44×10^−4^		70	–	–	–
IL4	30	4.08×10^6^	±9.75×10^3^	2.05×10^−6^	IL12	30	4.47×10^6^	±3.19×10^4^	2.30×10^−6^
	40	1.31×10^6^	±4.25×10^3^	6.41×10^−6^		40	3.47×10^6^	±2.51×10^4^	2.97×10^−6^
	50	3.64×10^5^	±1.03×10^3^	2.30×10^−5^		50	2.44×10^6^	±1.75×10^4^	4.21×10^−6^
	60	8.37×10^4^	±8.34×10^1^	1.00×10^−4^		60	1.39×10^6^	±1.25×10^4^	7.40×10^−6^
	70	1.42×10^4^	±7.04	5.91×10^−4^		70	6.30×10^5^	±6.82×10^3^	1.63×10^−5^
IL5	30	3.15×10^6^	±1.31×10^4^	2.48×10^−6^	IL13	30	1.39×10^6^	±1.48×10^4^	5.60×10^−6^
	40	5.80×10^6^	±6.68×10^4^	1.35×10^−6^		40	7.02×10^5^	±6.99×10^3^	1.11×10^−5^
	50	2.98×10^6^	±2.58×10^4^	2.62×10^−6^		50	4.85×10^5^	±5.38×10^3^	1.61×10^−5^
	60	3.55×10^5^	±6.75×10^2^	2.20×10^−5^		60	2.84×10^5^	±4.97×10^2^	2.74×10^−5^
	70	7.60×10^4^	±8.39×10^1^	1.03×10^−4^		70	1.18×10^5^	±3.55×10^2^	6.63×10^−5^
IL6	30	1.75×10^6^	±2.15×10^4^	5.63×10^−6^	IL14	30	2.23×10^5^	±3.72×10^3^	4.83×10^−5^
	40	5.68×10^5^	±3.39×10^4^	1.73×10^−5^		40	2.69×10^5^	±4.21×10^3^	4.00×10^−5^
	50	2.54×10^5^	±1.51×10^4^	3.88×10^−5^		50	2.94×10^5^	±4.71×10^3^	3.66×10^−5^
	60	1.45×10^5^	±4.34×10^3^	6.78×10^−5^		60	4.29×10^5^	±5.75×10^3^	2.51×10^−5^
	70	6.25×10^4^	±1.82×10^3^	1.57×10^−4^		70	3.25×10^5^	±4.79×10^3^	3.32×10^−5^
IL7	30	1.90×10^6^	±5.04×10^4^	4.41×10^−6^	IL15	30	2.21×10^6^	±1.29×10^4^	6.39×10^−6^
	40	1.00×10^6^	±2.22×10^4^	8.34×10^−6^		40	1.97×10^6^	±1.13×10^4^	7.19×10^−6^
	50	5.59×10^5^	±8.23×10^3^	1.50×10^−5^		50	7.97×10^5^	±3.56×10^3^	1.77×10^−5^
	60	2.51×10^5^	±3.29×10^3^	3.34×10^−5^		60	3.96×10^5^	±1.20×10^3^	3.57×10^−5^
	70	1.10×10^5^	±4.32×10^3^	7.60×10^−5^		70	1.19×10^5^	±2.02×10^2^	1.19×10^−4^
IL8	30	7.35×10^6^	±2.28×10^5^	1.54×10^−6^					
	40	3.33×10^6^	±6.26×10^4^	3.39×10^−6^					
	50	1.16×10^6^	±1.15×10^4^	9.78×10^−6^					
	60	4.79×10^5^	±4.63×10^3^	2.36×10^−5^					
	70	1.49×10^5^	±3.27×10^3^	7.58×10^−5^					

[a] Temperature. [b] Resistance of the bulk phase. [c] Resistance error. [d] Conductivity.

Besides these side reactions, **IL1** and **IL11** have the lowest melting points and show a broad melting peak, indicating first melting processes starting at temperatures as low as 55 °C. Because all measurements were conducted from 30 to 70 °C, this will affect the results because, in contrast to all other ILs studied here, **IL1** and **IL11** are partly liquid during these measurements. Furthermore, **IL2** shows very weak pre‐melting processes around 70 °C just before its melting point, which may slightly improve the MIL‐Electrode‐contact surface.

The temperature range was chosen for the following reasons: 1) the general melting temperatures of the MILs are between 64 and 101 °C, with all of them being above 70 °C with the exception of **IL1**, 2) operation temperatures of devices, for example, photovoltaic cells, are between 50 and 70 °C,^[62]^ and 3) chloride‐containing MILs have been studied in the same temperature range.

**Figure 9:**
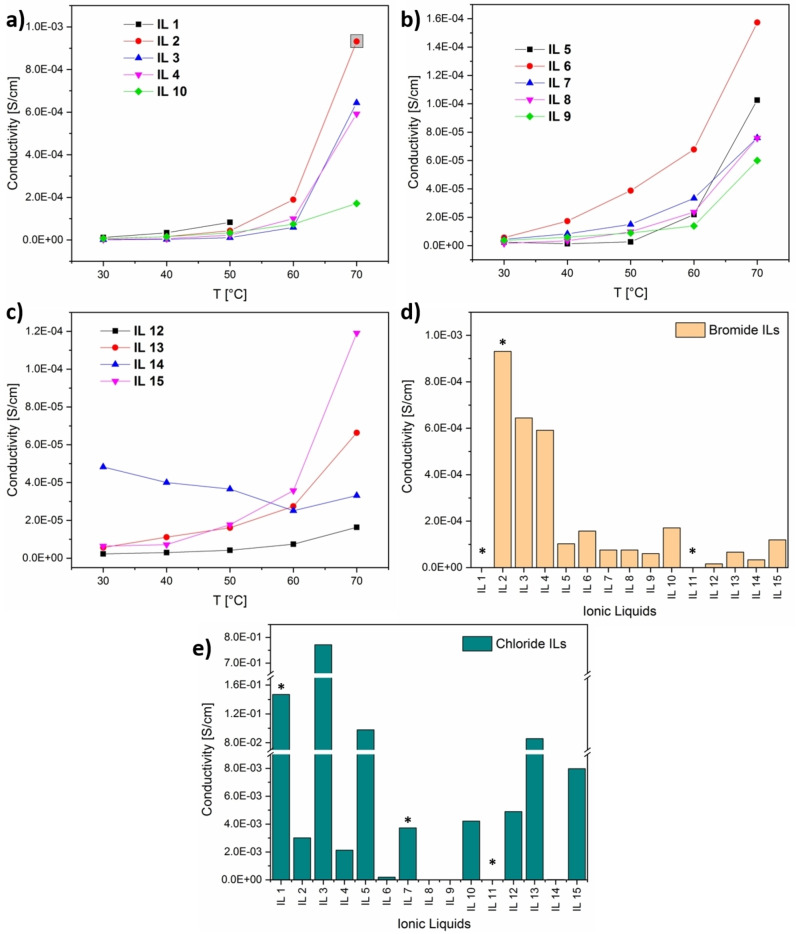
a)–c) Temperature‐dependent conductivity data of the bromide‐containing ILs (Table 1). d) Comparison of the conductivities of the bromide‐containing **IL1**–**15** at 70 °C. e) Comparison of conductivities of the corresponding chloride‐containing **IL1**–**15** at 70 °C. Note: Conductivity values could not be obtained at 60 and 70 °C for the bromide‐containing **IL1** or for either **IL11** variant due to the rather large scattering of the raw data points. Data points highlighted by a gray square in (a) and with a star in (d) and (e) indicate that these data were obtained right in the temperature window where a melting onset (start of the melting process) was already taking place. This can result in values that are higher than if the materials were pure solids. . Also note the different scales on the *y*‐axes in (d) and (e).

Generally, the temperature‐dependent measurements show that near room temperature the combined conductivity (ionic and electronic) of all ILs ranges between 10^−6^ and 10^−5^ S cm^−1^, with **IL14** showing the highest conductivity of 4.83 ⋅ 10^−5^ S cm^−1^. This is in agreement with the corresponding chloride‐containing ILs, which show conductivities in the range of 10^−4^ to 10^−8^ S cm^−1^ near room temperature (Figure 9a–c). Interestingly, the range of the conductivities observed for the bromide‐based systems is much smaller than in the case of the chloride‐based MILs.

At 70 °C the conductivities increase by up to two orders of magnitude (Table 5 and Figure 9d, e), with the exception of **IL14**, which shows a rather unusual behavior with a roughly constant conductivity. The resulting conductivities range between 10^−5^ and 10^−4^ S cm^−1^, with **IL2** showing the highest conductivity of 9.3×10^−4^ S cm^−1^.

Overall, with the exception of **IL14**, all MILs show the same general behavior, which is an increased conductivity versus temperature. This is expected as a temperature increase leads to a higher ionic mobility in the ILs and hence to a higher conductivity.

Generally, the conductivities of the current bromide‐containing MILs are comparable to other MILs, pyridinium‐based compounds, or eutectic mixtures, including the chloride analogues of the compounds presented here.[^42^, ^63^, ^64^]

It is interesting to note that overall the conductivities of the bromide‐based MILs are much more uniform when compared to the chloride analogues. In general the conductivity is lower, but the increase versus temperature is quite similar. In contrast, chloride‐based MILs show a much larger diversity in their conductivities versus temperature. This rather uniform conductivity versus temperature correlation suggest that the bromide‐containing MILs show slightly lower ionic mobilities but are more stable when applying a voltage.

This hypothesis is supported by the fact that no corrosion, decomposition or degradation processes can be observed during or after the measurements. This is in contrast to the chloride‐containing MILs, where some compounds show a highly corrosive behavior where the electrodes and the MILs are visibly changed after the measurements. Moreover, in these cases products of decomposition and degradation processes like CuCl can be found when working with chloride‐based MILs.[^23^, ^65^] In contrast, the current MILs never show such a behavior and all processes remain reversible with no signs of MIL decomposition. As a result, the higher conductivities of the more corrosive chloride‐based MILs might partially be caused by the formation and influence of CuCl.[^66^, ^67^, ^68^, ^69^]

As a result, the direct comparison of the chloride‐based^[42]^ and the bromide‐based MILs (this study) shows that there is a tradeoff between conductivity and corrosion: while from a conductivity (and cost) standpoint, the chloride‐based ILs would be attractive, they have also have a major drawback, which is the fact that they will corrode the electrodes in an electrochemical device rather rapidly. In fact, MILs are well known for their corrosive behavior.[^42^, ^70^, ^71^, ^72^]

As a result, chloride‐based MILs can corrode the gold plating of the electrodes, which exposes the underlying nickel layer, which is then oxidized by the transition metals of the [MCl_4_]^2−^ anion of the MILs or by the formation of Cl_2_ species in the course of the reaction.^[42]^ These Cl_2_ species can then attack the gold and also lead to the corrosion just described.

In contrast, MILs based on the [MBr_4_]^2−^ anion will not be able to do this as the redox potential is different from the redox potential of bromide (1.37 V for Cl_2_ vs. 1.07 V for Br_2_) Consequently, the current study shows that bromide‐based MILs have several advantages: 1) they are less corrosive and 2) they are more stable than the corresponding chlorides. Considering these advantages along with the fact that they are solid at room temperature, the current data suggest that the bromide‐containing MILs are highly promising materials for solid‐state electrolyte (SE) batteries[^73^, ^74^] or as electrolytes for applications at intermediate temperatures.^[75]^

The aforementioned tradeoff of the IL conductivity versus (electrochemical) IL stability is clearly the result of the halide change within the MIL structures. The main part contributing to the stability of the [MBr_4_]^2−^ compounds is the lower redox potential of bromide (1.37 V for Cl_2_ vs. 1.07 V for Br_2_), which is the result of a more ionic character of the chlorides. Following Pearson's hard and soft acids and base (HSAB) concept, Cl^−^ is classified as a hard base, while Br^−^ belongs to the (borderline) soft bases.^[76]^ Consequently, Cl^−^ has a higher affinity for ionic bonding, a higher charge density alongside a smaller ion size and a lower polarizability compared to Br^−^. We propose that this is one of the main factors for the corrosive behavior of the [MCl_4_]^2−^ MILs. Furthermore, Cl^−^ has a higher hydrogen bonding ability, since protons are classified as hard acids, which can be seen in the single‐crystal structures of the [CuX_4_]^2−^ compounds. Here, the [CuCl_4_]^2−^ MIL forms a chain‐like structure as the result of stronger hydrogen bonds, while the bromide analogue forms a rather dimer‐like structure. As a consequence, we assume that the ionic conductivity of the [MCl_4_]^2−^ MILs is higher compared to the bromide analogues, as the presence of the ion‐guiding chain structure supports ion movement.

## Conclusions

This article provides new insights into metal‐containing halide‐based ILs regarding the influence of chloride or bromide on structural, thermal, and electrochemical properties. Furthermore, this work validates the theoretical approach of using DFT calculations based on related compounds to analyze the structure of (metal‐containing) ILs, which is often a problem due to the unwillingness of ILs to crystallize. Also, concerning electrochemical properties, the data presented here show that, in general, although bromide‐based MILs show slightly worse conductivity data and slightly higher melting points, they are much more stable and show a relatively uniform behavior. As for the previously published chloride‐based MILs, the conductivities can be directly adjusted with regard to the metal composition. As a result, bromide‐containing MILs might well be even more promising as materials to be used in a variety of applications, such as batteries, electrolytes, solar cells, or sensors.

## Experimental Section

**Chemicals**: *N*‐Butylpyridinium bromide (Merck≥99.0 %), Co^II^‐bromide (VWR≥99.0 %), Zn^II^‐bromide (VWR≥99.0 %), Ni^II^‐bromide (VWR 99.0 %), propan‐2‐ol (VWR≥99.0 %), Mn^II^‐bromide (VWR≥99.0 %, anhydrous), Cu^II^‐bromide (VWR≥99.0 %), magnesium sulfate AnalaR NORMAPUR (VWR≥98.0 %), acetonitrile (VWR≥99.8 %, anhydrous), ferrocene (Alfa Aesar≥99.0 %), were used as received.

**Synthesis**: Synthesis followed the scheme described in ref. [55]. The synthetic procedure was the following: 0.5 g (2.31 mmol) of the metal salts along with the equimolar amount of *N*‐butylpyridinium bromide (BuPyBr) in the ratio of 1 : 2 (4.63 mmol) were dissolved in 10 mL of propan‐2‐ol. The mixture was heated to 110 °C and held at that temperature for 60 min under reflux conditions. If the reaction involved NiCl_2_, water was added to improve solubility and the reaction was held at 110 °C for 6 h under reflux conditions. Rotary evaporation was used to remove the solvent and additional water, and the compounds were then dried under vacuum (10^−3^ bar) for 12 h. The resulting ILs were directly used for analysis without purification. Generally, the reactions led to a yield between 91 and 97 %. The synthesis was repeated multiple times to show reproducibility. Crystallization of the ILs was done using different methods: 1) small amounts of the IL were dissolved in different solvents, for example, methanol, ethanol, propan‐2‐ol, acetonitrile or acetone. After several days or weeks crystals were formed under cooling or solvent evaporation and used for further structure analysis. 2) Crystals were taken directly from the bulk material and used for X‐ray crystal structure analysis.

**Infrared spectroscopy**: A Nicolet iS5 by Thermo Scientific was used for the IR measurements alongside an ID7 ATR‐attachment with a diamond crystal. The samples were measured using ATR mode between 4000 and 400 cm^−1^ with a resolution of 4 cm^−1^. All samples were measured as powders.

**Thermogravimetric analysis**: TGA was carried out on a Mettler Toledo TGA/DSC 3+ Thermogravimetric Analyzer (temperature range: 25 to 1000 °C, mass resolution: 1.0 μg) at a heating rate of 10 K min^−1^ under nitrogen flow in alumina crucibles.

**Crystal structure analysis**: The bulk of the compound (C_4_Py)_2_[CuBr_4_] was analyzed under an optical microscope, it is notable that the crystallinity of the compound is very low as it can be seen in Figure S1. Suitable crystals for X‐ray diffraction were separated by perfluorinated oil (Figure S1) mounted at the sample holder, and analyzed at a Stoe Stadivari diffractometer with Mo_Kα_ radiation (*λ*=0.71073 Å). The data were corrected using the program X‐Area and the structure was solved by direct methods and refined against *F* ^2^ on all data by full‐matrix least‐squares using the SHELX suite of programs.[^77^, ^78^] The crystal structure was visualized with Diamond 4.^[79]^

Deposition Number 2152368 contains the supplementary crystallographic data for this paper. These data are provided free of charge by the joint Cambridge Crystallographic Data Centre and Fachinformationszentrum Karlsruhe Access Structures service.

The Hirshfeld surface (HS) analysis was performed by using the program CrystalExplorer^[80]^ with the CIF of the compound (C_4_Py)_2_[CuBr_4_] as input file. The HS was calculated using a high surface resolution, the *d*_norm_ surface was mapped over a range from −0.07 (red) to 1.0 Å (blue). The red spots at the surface indicate close contacts between the atoms of the anion and atoms of the neighboring butyl‐pyridinium cations.

**Powder X‐ray diffraction**: X‐ray powder diffraction data were collected on a PANalytical Empyrean powder X‐ray diffractometer in a Bragg–Brentano geometry using Cu_Kα_ radiation (*λ*=1.5419 Å). It was equipped with a programmable divergence and anti‐scatter slit and a large Ni‐beta filter. The PIXcel1D detector was set to continuous mode with an active length of 3.0061°. *θ*/*θ* scans were run in a 2*θ* range of 4–70° with step size of 0.0131° operating at 40 kV and 40 mA. A sample rotation time of 1 s was used. The data set was recorded over 23 min for **IL1**–**IL15** and over 115 min (5 repetitions) for **IL13**.

**Differential scanning calorimetry**: DSC measurements were done using a Netzsch DSC 214 Polyma at 5–10 K min^−1^ under nitrogen. Each run consisted of three heating‐cooling cycles.

**Electrochemistry**: Impedance spectra were measured as follows: An impedance setup of the Autolab PGSTAT204 system (Metrohm) was used to measure from 10^5^ Hz to 1 Hz with an amplitude of 0.3 V. A temperature‐controlled microcell HC (rhd instruments) was used for temperature‐dependant measurements, with the measurements carried out at temperatures ranging from 25 to 70 °C. A Peltier element with an accuracy of 0.01 °C conducted heating and cooling. The compounds were prepared as follows: The ILs were placed in separate silicon forms and heated to 110 °C in a heating oven, with the temperature then being held for 60 min. Besides removing most of the residual water, this process produced a macroscopic pellet for usage in the measuring cell. This also ensures a large contact area with the electrodes. The pellets were then placed between the electrodes in the measuring cell. The conductivity *σ* was calculated from Equation (1), with *R* being the (electrolyte) resistance, *A* being the surface content of the circular‐shaped electrodes, and *d* being the height of the sample, which also is the thickness of the electrode layer.

(1) $$\sigma =\;\frac{d}{R\;\cdot \;A}$$

Cyclic voltammetry (CV) measurements were conducted as follows: A three‐electrode system of a Metrohm Autolab PGSTAT204 was used to perform the measurements. The ILs (0.1 M) were solved in a dry acetonitrile/ferrocene solution, which was then placed in the measurement chamber. A Pt counter electrode with a surface of 1 cm^2^ (with a central hole for reference electrode), a silver reference electrode with a diameter of 2 mm, and a glassy carbon working electrode were inserted. The ferrocene (5×10^−4^ M) was used as a reference.

**Electrode and cell assembly**: During the impedance measurements, the performance of the pure ILs was analyzed using a two‐electrode cell setup (Pt electrode, graphite electrode) with a rhd TSC battery (Ni electrodes 9 mm) in combination with a Metrohm Autolab PGSTAT204. To prevent corrosion and degradation of the Ni electrodes, Au electrodes were placed between sample and Ni electrodes. Measurements were done using the NOVA software (Metrohm). Analysis was done using the RelaxIS software (rhd instruments, Marburg, Germany).

**Inductively coupled plasma optical emission spectroscopy**: ICP‐OES measurements were conducted as follows: The measurements were performed using the PerkinElmer Optical Spectrometer 5300 DV with the cyclon chamber/MiraMist nebulizer. Read time was 2–10 s with a power of 1400 W and plasma gas flow of 17 L min^−1^, nebulizer gas flow of 0.6 L min^−1^ and auxiliary gas flow of 0.2 L min^−1^. The measurements were performed axially. All samples were dissolved in HNO_3_ (3 %) before the measurement. The wavelengths were as follows: Cu 327.393 nm, Co 238.892 nm, Mn 257.610 nm, Ni 231.604 nm and Zn 206.200 nm.

**DFT simulations**: All calculations were done using the Vienna Ab initio Simulation Package[^81^, ^82^] using the PBE functional^[83]^ and a projector‐augmented plane‐wave basis (PAW).[^84^, ^85^] Dispersion forces are included using Grimmes D3 method^[86]^ with Becke‐Jonson damping^[87]^ (IVDW=12). Further, we include non‐spherical contributions from the gradient corrections inside the PAW spheres (LASPH=.TRUE.). Based on convergence tests we used spin polarized calculations with cut‐off energies of 400 eV and Gamma point only for the refinement of the geometries. Further, the energetic difference between the ferromagnetic and different antiferromagnetic densities is extremely small. In what follows we use an initial density, which corresponds to 5.5 unpaired electrons on each Mn, by using the keyword “Magmon”.

For all calculations, the structure model was taken from the crystallographic data of the monometallic chlorido **MIL11**. The initial lattice parameters were 14.6, 16.9 and 16.0 A.

The powder X‐ray diffractograms were simulated with the pattern fitting system based on the maximum‐entropy method of RIETAN‐2000.^[88]^

## Conflict of interest

The authors declare no conflict of interest.

1

## Supporting information

As a service to our authors and readers, this journal provides supporting information supplied by the authors. Such materials are peer reviewed and may be re‐organized for online delivery, but are not copy‐edited or typeset. Technical support issues arising from supporting information (other than missing files) should be addressed to the authors.

Supporting Information

## Data Availability

The data that support the findings of this study are available in the supplementary material of this article.

## References

[1] M. Armand , F. Endres , D. R. MacFarlane , H. Ohno , B. Scrosati , Nat. Mater. 2009, 8, 621–629. 19629083 10.1038/nmat2448

[2] T. Torimoto , T. Tsuda , K. I. Okazaki , S. Kuwabata , Adv. Mater. 2010, 22, 1196–1221. 20437507 10.1002/adma.200902184

[3] J. P. Hallett , T. Welton , Chem. Rev. 2011, 111, 3508–3576. 21469639 10.1021/cr1003248

[4] K. R. Seddon , J. Chem. Technol. Biotechnol. 1997, 68, 351–356.

[5] M. Freemantle, *An Introduction to Ionic Liquids*, Royal Society of Chemistry, **2009**.

[6] D.-Y. Wang , C.-Y. Wei , M.-C. Lin , C.-J. Pan , H.-L. Chou , H.-A. Chen , M. Gong , Y. Wu , C. Yuan , M. Angell , Y.-J. Hsieh , Y.-H. Chen , C.-Y. Wen , C.-W. Chen , B.-J. Hwang , C.-C. Chen , H. Dai , Nat. Commun. 2017, 8, 14283. 28194027 10.1038/ncomms14283 PMC5316828

[7] J. Ding , D. Zhou , G. Spinks , G. Wallace , S. Forsyth , M. Forsyth , D. MacFarlane , Chem. Mater. 2003, 15, 2392–2398.

[8] P. Wang , S. M. Zakeeruddin , I. Exnar , M. Grätzel , Chem. Commun. 2002, 5, 2972–2973. 10.1039/b209322g 12536772

[9] F. Fabregat-Santiago , J. Bisquert , E. Palomares , L. Otero , D. Kuang , S. M. Zakeeruddin , M. Grätzel , J. Phys. Chem. C 2007, 111, 6550–6560.

[10] A. Abouserie , K. Zehbe , P. Metzner , A. Kelling , C. Günter , U. Schilde , P. Strauch , T. Körzdörfer , A. Taubert , Eur. J. Inorg. Chem. 2017, 2017, 5640–5649.

[11] A. Angeloni , A. G. Orpen , Chem. Commun. 2001, No. 4, 343–344.

[12] M. W. Bouwkamp , E. Lobkovsky , P. J. Chirik , J. Am. Chem. Soc. 2005, 127, 9660–9661. 15998050 10.1021/ja0524447

[13] G. R. Lewis , Chem. Commun. 1998, 1873–1874.

[14] C. K. Lee , K.-M. Hsu , C.-H. Tsai , C. K. Lai , I. J. B. Lin , Dalton Trans. 2004, 4, 1120. 10.1039/b314367h 15252650

[15] C. K. Lee , M. J. Ling , I. J. B. Lin , Dalton Trans. 2003, 1, 4731–4737.

[16] W. A. Herrmann , L. J. Goossen , G. R. J. Artus , C. Köcher , Organometallics 1997, 16, 2472–2477.

[17] K. Zehbe , M. Kollosche , S. Lardong , A. Kelling , U. Schilde , A. Taubert , Int. J. Mol. Sci. 2016, 17, 391. 26999112 10.3390/ijms17030391 PMC4813247

[18] R. Giernoth , Angew. Chem. Int. Ed. 2010, 49, 2834–2839; 10.1002/anie.200905981 20229544

[19] A. Taubert , Z. Li , Dalton Trans. 2007, No. 7, 723–727. 10.1039/b616593a 17279241

[20] A. P. Abbott , T. J. Bell , S. Handa , B. Stoddart , Green Chem. 2005, 7, 705.

[21] B. Dolling , A. L. Gillon , A. G. Orpen , J. Starbuck , X.-M. Wang , Chem. Commun. 2001, 567–568.

[22] R. Kore , S. P. Kelley , P. Aduri , R. D. Rogers , Dalton Trans. 2018, 47, 7795–7803. 29850701 10.1039/c8dt00976g

[23] A. Taubert , Angew. Chem. Int. Ed. 2004, 43, 5380–5382; 10.1002/anie.200460846 15468093

[24] A. Taubert , Acta Chim. Slov. 2005, 52, 168–170.

[25] A. Taubert , Acta Chim. Slov. 2005, 52, 183–186.

[26] M. Antonietti , D. Kuang , B. Smarsly , Y. Zhou , Angew. Chem. Int. Ed. 2004, 43, 4988–4992; 10.1002/anie.200460091 15372641

[27] J. Richter , M. Ruck , Molecules 2019, 25, 78. 31878305 10.3390/molecules25010078 PMC6983208

[28] A. Abouserie , G. A. El-Nagar , B. Heyne , C. Günter , U. Schilde , M. T. Mayer , S. Stojkovikj , C. Roth , A. Taubert , Appl. Mater. Interf. 2020, 12, 52560–52570. 10.1021/acsami.0c13927 33180455

[29] Y. Kim , B. Heyne , A. Abouserie , C. Pries , C. Ippen , C. Günter , A. Taubert , A. Wedel , J. Chem. Phys. 2018, 148. 10.1063/1.4991622 30307196

[30] F. Neve , A. Crispini , S. Armentano , O. Francescangeli , Chem. Mater. 1998, 10, 1904–1913.

[31] F. Neve , O. Francescangeli , A. Crispini , Inorg. Chim. Acta 2002, 338, 51–58.

[32] F. Neve , O. Francescangeli , A. Crispini , J. Charmant , Chem. Mater. 2001, 13, 2032–2041.

[33] F. Neve , M. Impéror-Clerc , Liq. Cryst. 2004, 31, 907–912.

[34] C. J. Bowlas , D. W. Bruce , K. R. Seddon , Chem. Commun. 1996, 14, 1625.

[35] J. Estager , J. D. Holbrey , M. Swadźba-Kwaśny , Chem. Soc. Rev. 2014, 43, 847–886. 24189615 10.1039/c3cs60310e

[36] A. G. Zazybin , K. Rafikova , V. Yu , D. Zolotareva , V. M. Dembitsky , T. Sasaki , Russ. Chem. Rev. 2017, 86, 1254–1270.

[37] C. Chiappe , M. Malvaldi , Phys. Chem. Chem. Phys. 2010, 12, 11191. 20657911 10.1039/c001796e

[38] K. Goossens , K. Lava , P. Nockemann , K. Van Hecke , L. Van Meervelt , P. Pattison , K. Binnemans , T. Cardinaels , Langmuir 2009, 25, 5881–5897. 19374454 10.1021/la900048h

[39] D. Kim , Y. Moon , D. Ji , H. Kim , D. Cho , ACS Sustainable Chem. Eng. 2016, 4, 4591–4600.

[40] Y. Liu , J. Wang , Molecules 2018, 23, 2516.30274390

[41] Y. Yoshida , G. Saito , J. Mater. Chem. 2006, 16, 1254.

[42] C. Balischewski , K. Behrens , K. Zehbe , C. Günter , S. Mies , E. Sperlich , A. Kelling , A. Taubert , Chem. Eur. J. 2020, 26, 17504–17513. 32841435 10.1002/chem.202003097 PMC7839689

[43] S. Men , D. S. Mitchell , K. R. J. Lovelock , P. Licence , ChemPhysChem 2015, 16, 2211–2218. 25952131 10.1002/cphc.201500227 PMC4768647

[44] R. Farra , K. Thiel , A. Winter , T. Klamroth , A. Pöppl , A. Kelling , U. Schilde , A. Taubert , P. Strauch , New J. Chem. 2011, 35, 2793.

[45] A. Winter , K. Thiel , A. Zabel , T. Klamroth , A. Pöppl , A. Kelling , U. Schilde , A. Taubert , P. Strauch , New J. Chem. 2014, 38, 1019.

[46] M. A. Spackman , D. Jayatilaka , CrystEngComm 2009, 11, 19–32.

[47] A. N. Usoltsev , S. A. Adonin , P. E. Plyusnin , P. A. Abramov , I. V. Korolkov , M. N. Sokolov , V. P. Fedin , Polyhedron 2018, 151, 498–502.

[48] D. Quiñonero , C. Garau , C. Rotger , A. Frontera , P. Ballester , A. Costa , P. M. Deyà , Angew. Chem. Int. Ed. 2002, 41, 3389–3392; 10.1002/1521-3773(20020916)41:18<3389::AID-ANIE3389>3.0.CO;2-S 12298041

[49] K. R. Seddon , A. Stark , M.-J. Torres , Pure Appl. Chem. 2000, 72, 2275–2287.

[50] M. Imanari , K. Fujii , T. Endo , H. Seki , K. Tozaki , K. Nishikawa , J. Phys. Chem. B 2012, 116, 3991–3997. 22409655 10.1021/jp300722j

[51] R. Bhattacharya , M. Sinha Ray , R. Dey , L. Righi , G. Bocelli , A. Ghosh , Polyhedron 2002, 21, 2561–2565.

[52] M. Ross , R. Boehler , D. Errandonea , Phys. Rev. B 2007, 76, 184117. 10.1103/PhysRevLett.85.3444 11030917

[53] M. C. M. O'Brien , C. C. Chancey , Am. J. Phys. 1993, 61, 688–697.

[54] H. A. Jahn , E. Teller , Proc. R. Soc. London Ser. A Math. Phys. Sci. 1937, 161, 220–235.

[55] K. Thiel , T. Klamroth , P. Strauch , A. Taubert , Phys. Chem. Chem. Phys. 2011, 13, 13537. 21617795 10.1039/c1cp20648f

[56] A. M. O'Mahony , D. S. Silvester , L. Aldous , C. Hardacre , R. G. Compton , J. Chem. Eng. Data 2008, 53, 2884–2891.

[57] K. Funke , Impedanzspektroskopie: Apparative Methoden in der Physikalischen Chemie, Vorlesungsskript, Universität Münster, 2002.

[58] D. Ende , K. Mangold , Chem. Unserer Zeit 1993, 134–140.

[59] E. Barsoukov, J. R. Macdonald. *Impedance Spectroscopy Theory, Experiment, and Applications*, Wiley, Hoboken, **2005**.

[60] S. Lanfredi , A. C. M. Rodrigues , J. Appl. Phys. 1999, 86, 2215–2219.

[61] D. U. Sauer , Tech. Mitt. 2015, 99, 7–11.

[62] L. Zhu , A. Raman , K. X. Wang , M. A. Anoma , S. Fan , Optica 2014, 1, 32.

[63] M. Watanabe , S. Yamada , N. Ogata , Electrochim. Acta 1995, 40, 2285–2288.

[64] I. Bandrés , D. F. Montaño , I. Gascón , P. Cea , C. Lafuente , Electrochim. Acta 2010, 55, 2252–2257.

[65] N. Garro , A. Cantarero , M. Cardona , T. Ruf , A. Göbel , C. Lin , K. Reimann , S. Rübenacke , M. Steube , Solid State Commun. 1996, 98, 27–30.

[66] J. Maier , Solid State Ionics 1987, 23, 59–67.

[67] J. Maier , J. Phys. Chem. Solids 1985, 46, 309–320.

[68] J. Maier , Prog. Solid State Chem. 1995, 23, 171–263.

[69] T. Jow , J. Electrochem. Soc. 1979, 126, 1963.

[70] H. Liu , Y. Liu , J. Li , Phys. Chem. Chem. Phys. 2010, 12, 1685. 20145833 10.1039/b921469k

[71] R.-S. Kühnel , N. Böckenfeld , S. Passerini , M. Winter , A. Balducci , Electrochim. Acta 2011, 56, 4092–4099.

[72] S. Menne , R.-S. Kühnel , A. Balducci , Electrochim. Acta 2013, 90, 641–648.

[73] M. Feinauer , H. Euchner , M. Fichtner , M. A. Reddy , ACS Appl. Energ. Mater. 2019, 2, 7196–7203.

[74] I. Mohammad , R. Witter , M. Fichtner , M. A. Reddy , ACS Appl. Energ. Mater. 2019, 2, 1553–1562.

[75] “Sodium-Metal Halide Batteries in Diesel-Battery Hybrid Telecom Applications”, J. Rijssenbeek, H. Wiegman, D. Hall, C. Chuah, G. Balasubramanian, C. Brady in *2011 IEEE 33rd International Telecommunications Energy Conference (INTELEC)*, IEEE, **2011**, pp. 1–4.

[76] R. G. Pearson , J. Chem. Educ. 1968, 45, 581.

[77] G. M. Sheldrick , Acta Crystallogr. Sect. A Found. Crystallogr. 2008, 64, 112–122. 10.1107/S0108767307043930 18156677

[78] G. M. Sheldrick , Acta Crystallogr. Sect. C Struct. Chem. 2015, 71, 3–8. 25567568 10.1107/S2053229614024218 PMC4294323

[79] K. Brandenburg , H. Putz , Diamond: Crystal and Molecular Structure Visualization, Crystal Impact Diamond, Bonn, 2020.

[80] P. R. Spackman , M. J. Turner , J. J. McKinnon , S. K. Wolff , D. J. Grimwood , D. Jayatilaka , M. A. Spackman , J. Appl. Crystallogr. 2021, 54, 1006–1011. 34188619 10.1107/S1600576721002910 PMC8202033

[81] G. Kresse , J. Furthmüller , Phys. Rev. B 1996, 54, 11169–11186. 10.1103/physrevb.54.11169 9984901

[82] G. Kresse , J. Furthmüller , Comput. Mater. Sci. 1996, 6, 15–50.

[83] J. P. Perdew , K. Burke , M. Ernzerhof , Phys. Rev. Lett. 1996, 77, 3865–3868. 10062328 10.1103/PhysRevLett.77.3865

[84] P. E. Blöchl , Phys. Rev. B 1994, 50, 17953–17979. 10.1103/physrevb.50.17953 9976227

[85] G. Kresse , D. Joubert , Phys. Rev. B 1999, 59, 1758–1775.

[86] S. Grimme , J. Antony , S. Ehrlich , H. Krieg , J. Chem. Phys. 2010, 132, 154104. 20423165 10.1063/1.3382344

[87] S. Grimme , S. Ehrlich , L. Goerigk , J. Comput. Chem. 2011, 32, 1456–1465. 21370243 10.1002/jcc.21759

[88] F. Izumi , K. Momma , Solid State Phenom. 2007, 130, 15–20.

