# Supplementary material for: Tetrahalidometallate(II) Ionic Liquids with More than One Metal: The Effect of Bromide versus Chloride
Source: Chemistry. 2022 Sep 15;28(64):e202201068. doi: 10.1002/chem.202201068 (PMC9826293; doi:10.1002/chem.202201068)
Supplement: Supplementary file 1 — Supporting Information [file CHEM-28-0-s001.pdf]

Chemistry–A European Journal

Supporting Information

Tetrahalidometallate(II) Ionic Liquids with More than One Metal: The Effect of Bromide versus Chloride

Christian Balischewski, Biswajit Bhattacharyya, Eric Sperlich, Christina Günter, Alkit Beqiraj, Tillmann Klamroth, Karsten Behrens, Stefan Mies, Alexandra Kelling, Susanne Lubahn, Lea Holtzheimer, Anne Nitschke, and Andreas Taubert*

Table of Contents

1. Crystal Structure Analysis 2

a. Detail of the Hirshfeld surface analysis: 2

2. Additional XRD data 3

3. Thermal Properties 5

a. Thermogravimetric Analysis 5

b. Differential Scanning Calorimetry 6

4. Cyclic Voltammetry 10

5. Special Case: $(BuPy)_2[Cu_{0.25}Co_{0.75}Br_4]$ 11

a. ICP-OES 11

b. Thermal Properties 11

i. Differential Scanning Calorimetry 11

ii. Thermal Gravimetric Analysis 12

c. Conductivity 13

1. Crystal Structure Analysis

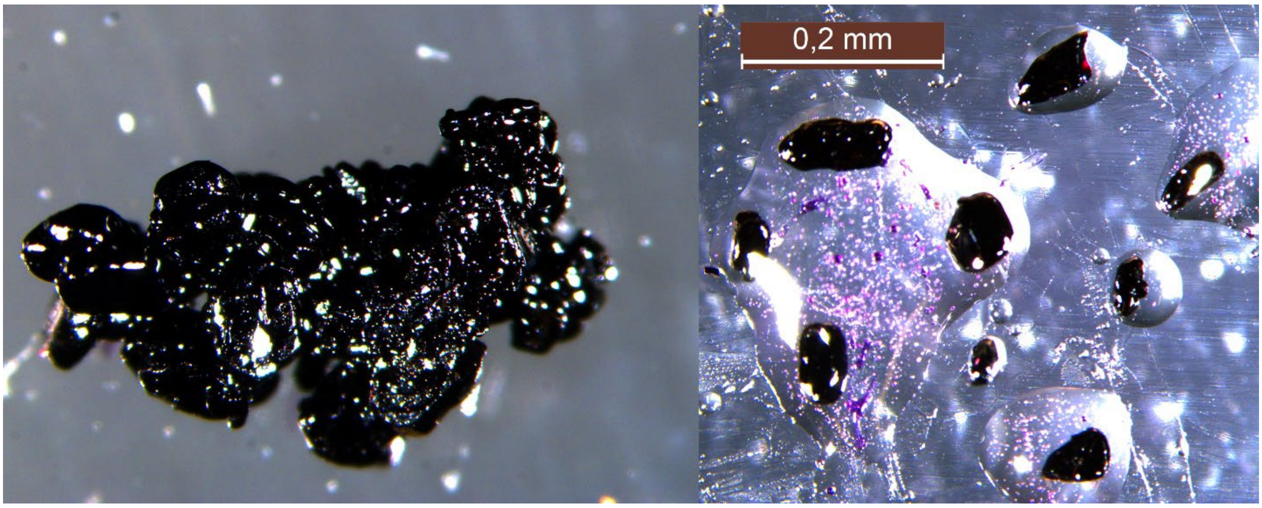

Figure S1. *Microscope images of the bulky solid (left) and some crystals of $(C_4\text{-Pyr})_2[CuBr_4]$ (right).*

a. Detail of the Hirshfeld surface analysis:

The Hirshfeld surface (HS) analysis was performed using the program CrystalExplorer with the CIF of the compound $(C_4\text{-Pyr})_2[CuBr_4]$ as input file. The HS was calculated using a high surface resolution, the d_{norm} surface was mapped over a range from -0.07 (red) to 1.0 Å (blue). The red spots at the surface indicate close contacts between the atoms of the anion and atoms of the neighboring butyl-pyridinium cations.

2. Additional XRD data

Additional X-ray measurements were done to study the contribution of remaining precursor material to structure of the synthesized MILs.

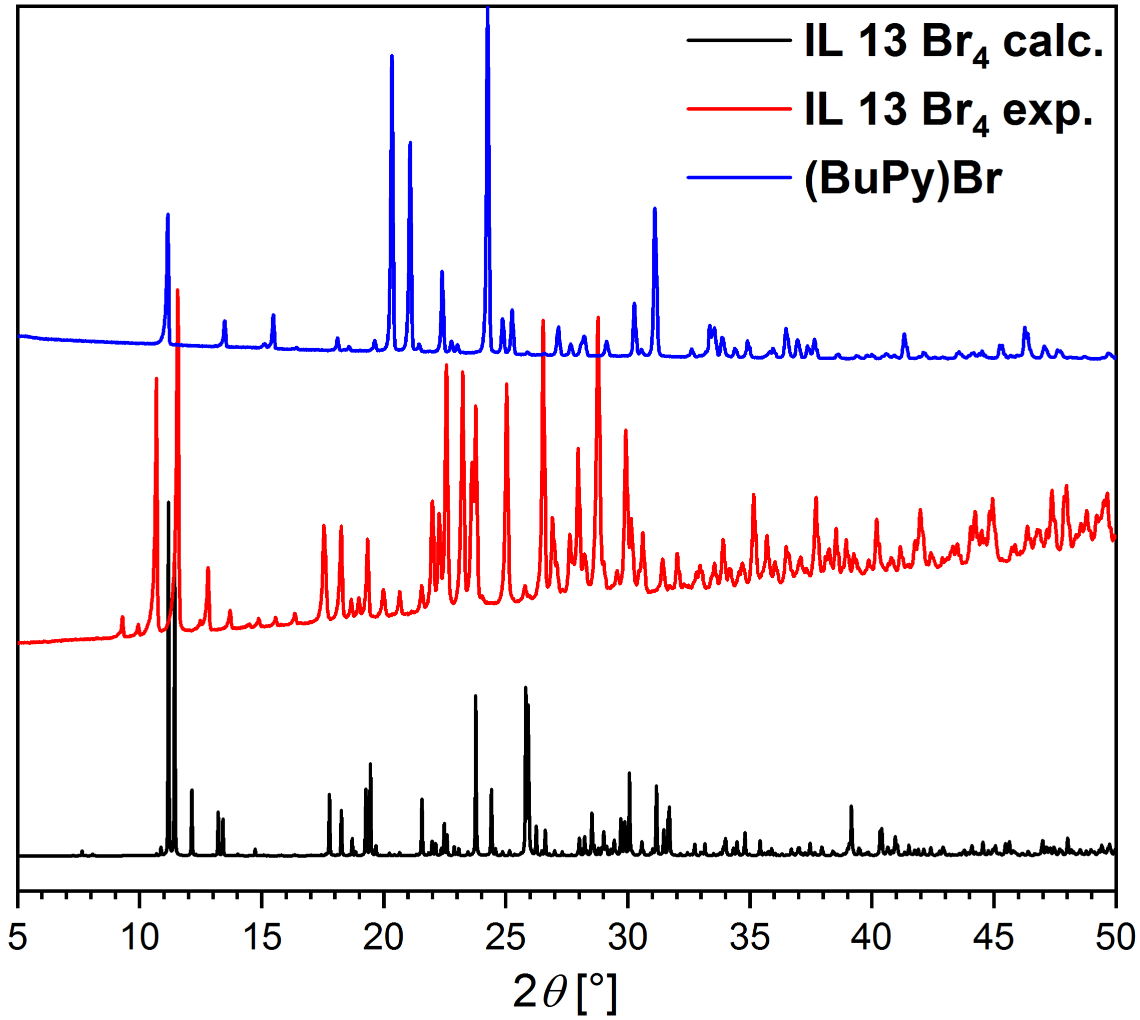

Figure S2. Comparison of the calculated X-ray diffractograms of **IL 13**, the experimental X-ray diffractograms of **IL 13** and the experimental diffractograms of the IL precursor (BuPy)Br.

Table S1. *Further d values of the corresponding lattice planes.*

**[h k l]**	***d* IL13 – Br_4 [Å]**	***d* IL13 – Cl_4 [Å]**
[0 0 2]	7.9	7.8
[1 2 -1]	7.7	7.6
[2 0 0]	7.3	7.1
[0 4 0]	4.6	4.6
[3 2 -1]	4.5	4.4
[1 4 2]	3.7	3.7
[2 2 3]	3.4	3.4

Table S2. *Comparison of the cell parameters of the different manganes-containing ILs.*

**IL 13 – Br_4**		**IL 13 – Cl_4**	
a	15.62 Å	a	15.20 Å
b	18.96 Å	b	18.50 Å
c	16.95 Å	c	16.70 Å
α	90.0	α	90.0
β	111.1	β	111.1
γ	90.0	γ	90.0
V	4682 Å³	V	4382 Å³

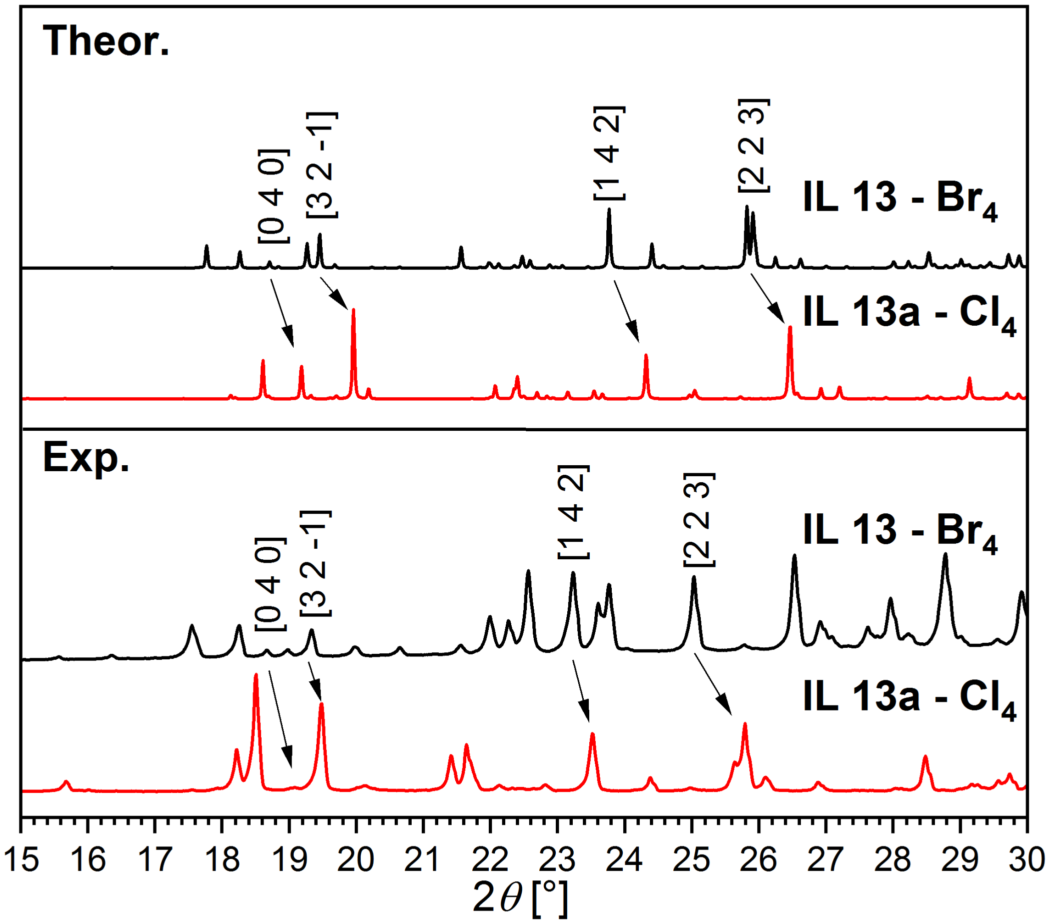

Figure S3. Detailed comparison of the calculated and experimental X-ray diffractograms of **IL 13** in the region of 15 to 30° 2*θ* with the corresponding *hkl* values.

3. Thermal Properties

Additional measurements were conducted to analyze thermal behavior, such as thermal decomposition, melting or crystallization processes.

a. Thermogravimetric Analysis

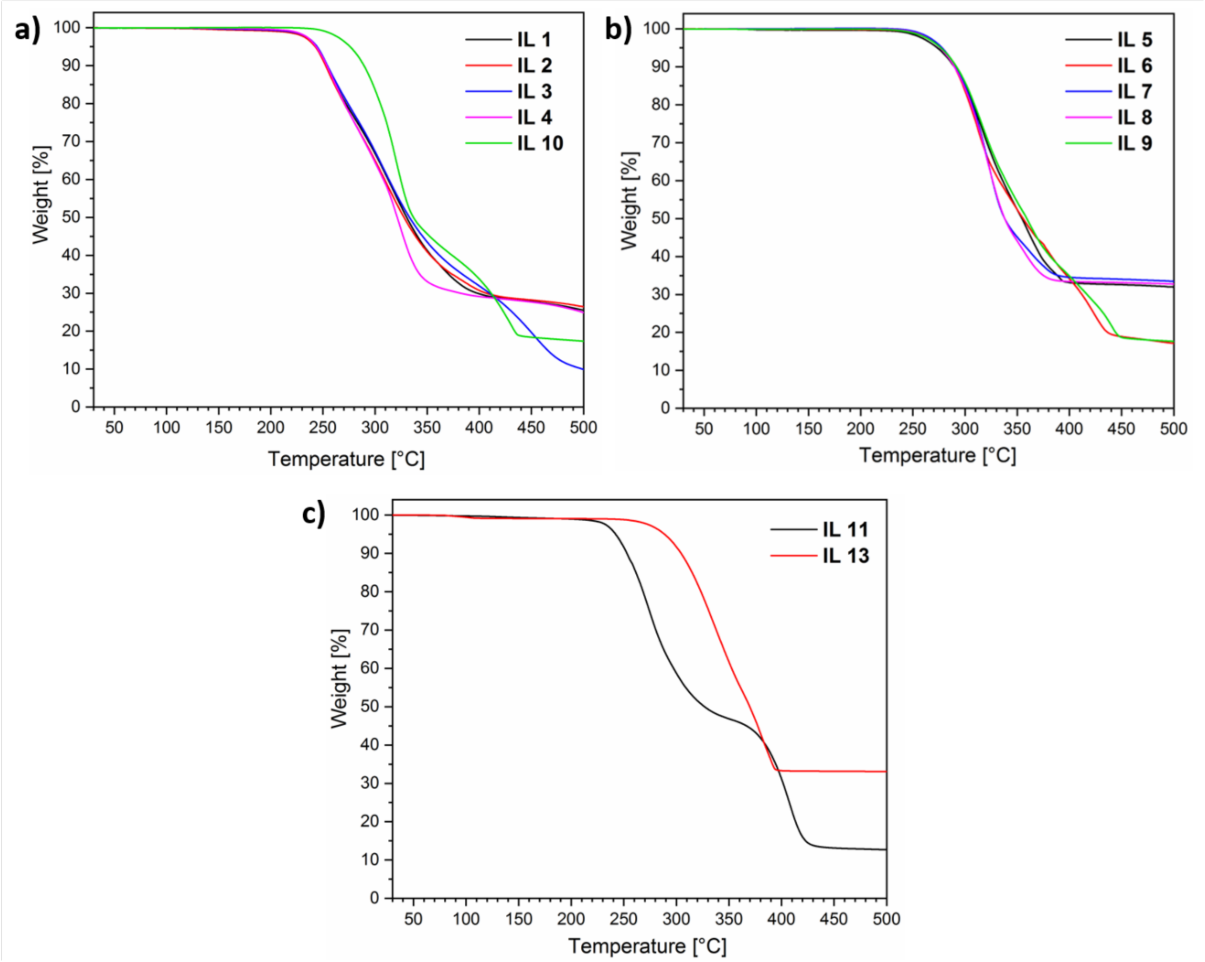

Figure S3. Comparison of the thermogravimetric data on a,b) bimetallic bromide-containing MILs and c) monometallic bromide-containing MILs.

b. Differential Scanning Calorimetry

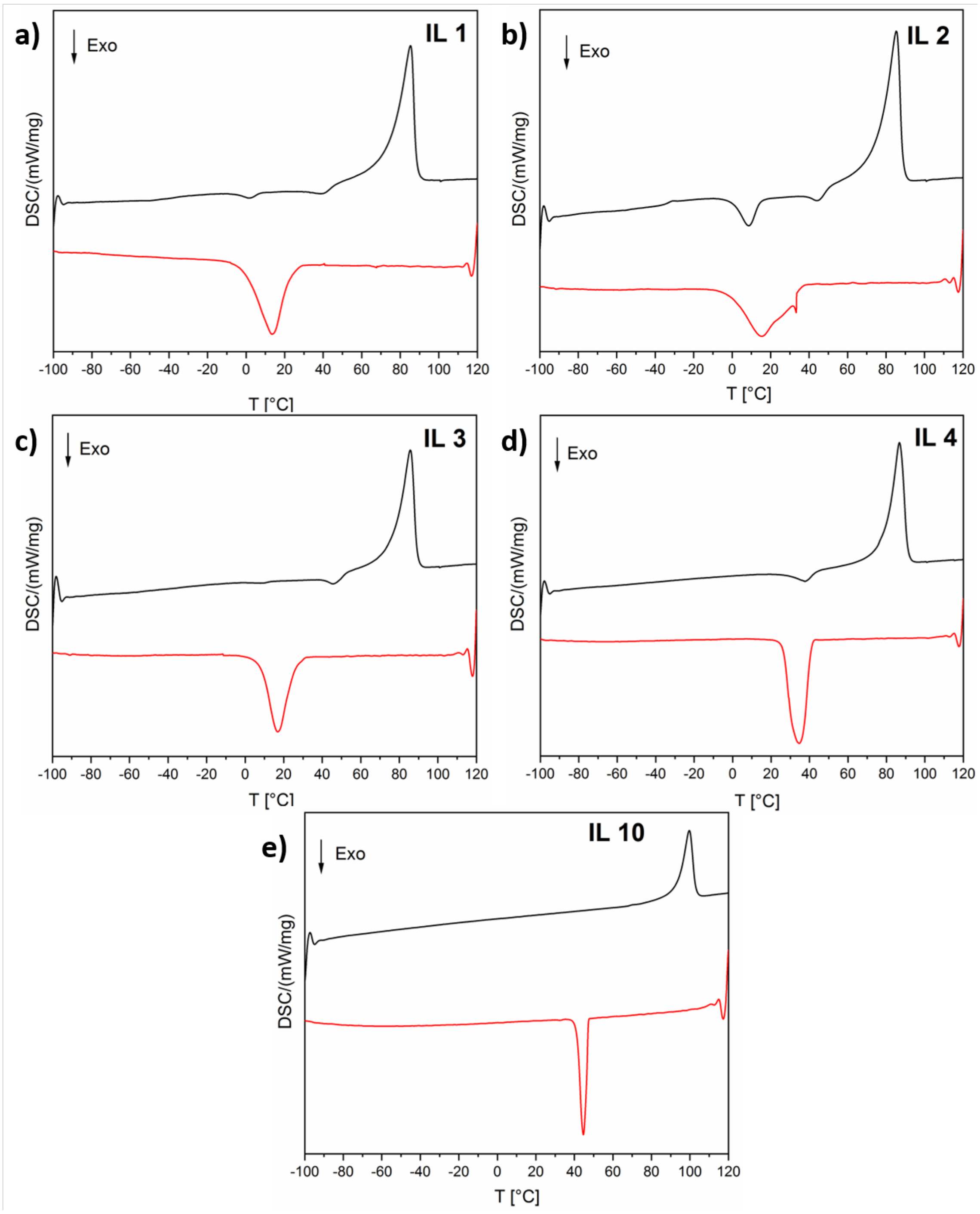

Figure S4. Comparison of the differential scanning calorimetry data on bimetallic bromide-containing **ILs 1-4** and **IL 10**.

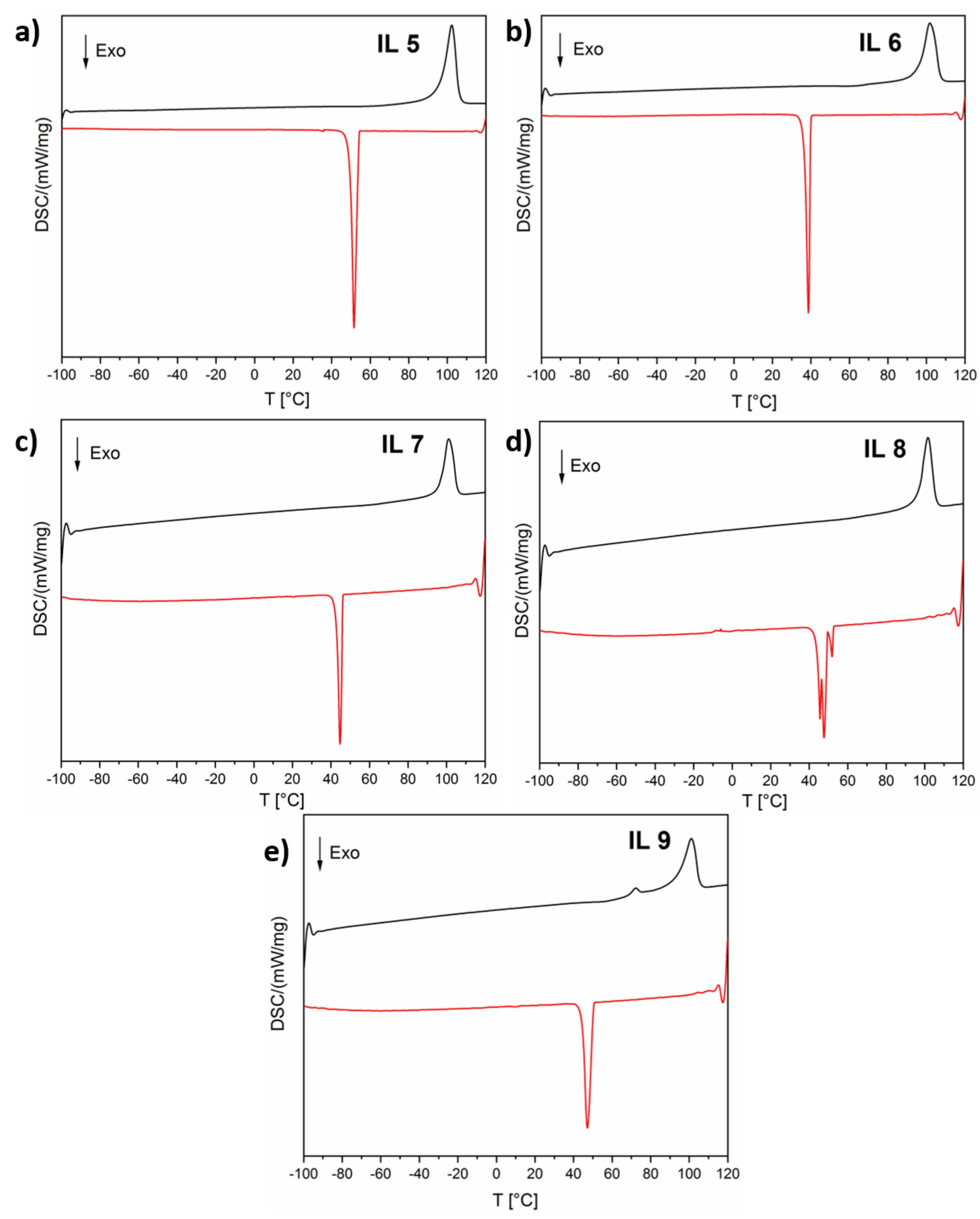

Figure S5. Comparison of the differential scanning calorimetry data on bimetallic bromide-containing **ILs 5-9**.

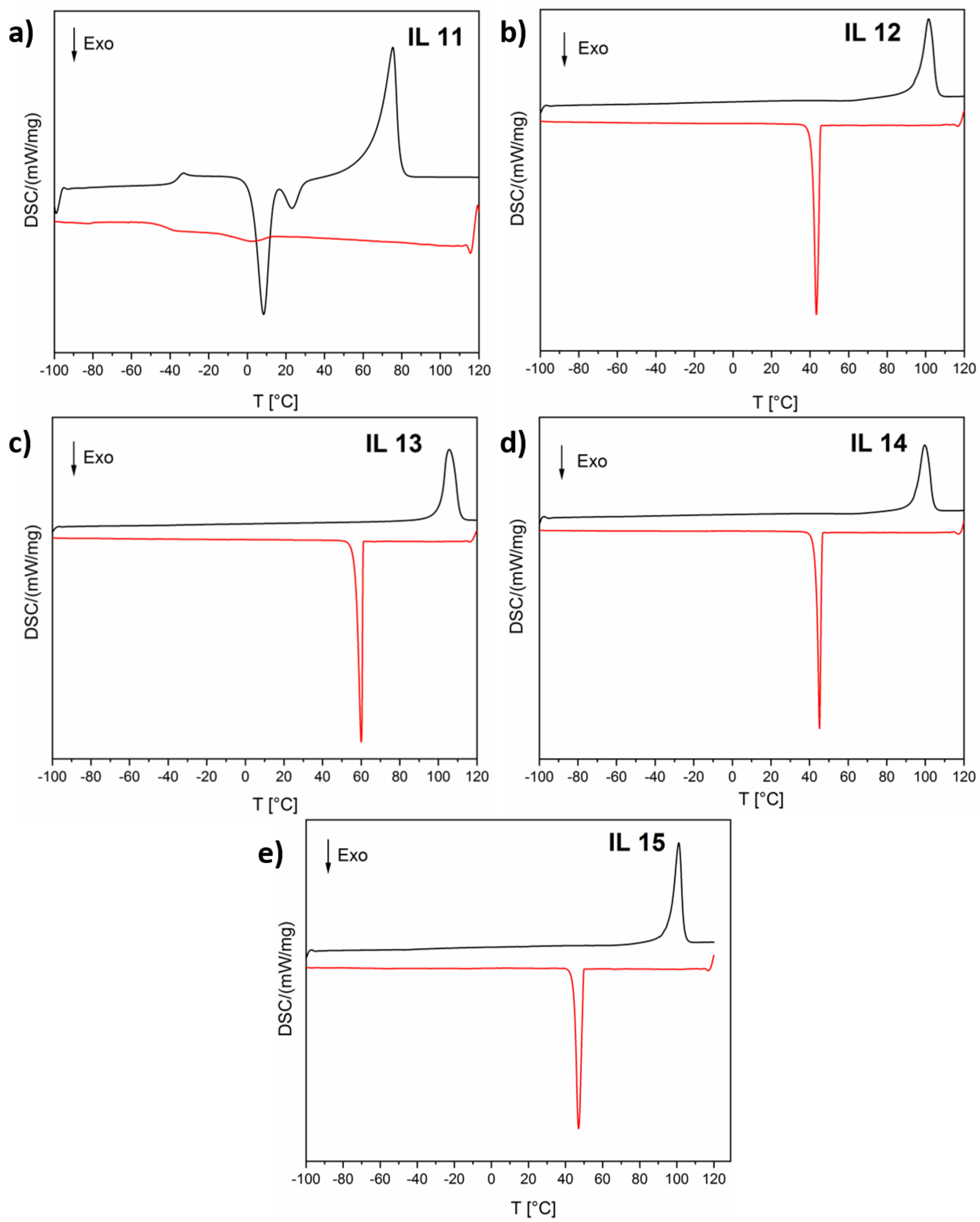

Figure S6. Comparison of the differential scanning calorimetry data on monometallic bromide-containing **ILs 11-15**.

Table S 3. *Melting transitions extracted from DSC heating curves (IL 1-15)*

Compound	Run	T_m [°C]	Δ*H* [J/g]	Δ*H* [kJ/mol]
**IL 1**	2nd	73.3	42.36	27.59
	3rd	73.5	41.64	27.12
**IL 2**	2nd	74.4	41.32	26.99
	3rd	74.3	40.10	26.20
**IL 3**	2nd	76.1	42.83	28.12
	3rd	76.0	42.33	27.79
**IL 4**	2nd	78.7	48.18	31.47
	3rd	78.4	46.93	30.65
**IL 5**	2nd	95.7	52.44	34.03
	3rd	95.8	52.42	34.02
**IL 6**	2nd	95.6	50.97	33.34
	3rd	95.7	51.35	33.59
**IL 7**	2nd	96.3	54.73	35.62
	3rd	96.3	53.95	35.11
**IL 8**	2nd	96.2	55.82	36.22
	3rd	95.9	56.60	36.73
**IL 9**	2nd	93.1	45.56	29.71
	3rd	92.8	45.95	29.97
**IL 10**	2nd	93.1	50.83	33.25
	3rd	93.2	51.63	33.77
**IL 11**	2nd	64.7	40.59	26.61
	3rd	64.7	40.43	26.51
**IL 12**	2nd	95.5	52.64	34.27
	3rd	95.4	52.60	34.24
**IL 13**	2nd	101.0	57.84	37.42
	3rd	99.7	57.36	37.11
**IL 14**	2nd	94.1	53.33	34.70
	3rd	94.2	54.41	35.41
**IL 15**	2nd	95.9	52.72	34.66
	3rd	96.0	52.80	34.71

4. Cyclic Voltammetry

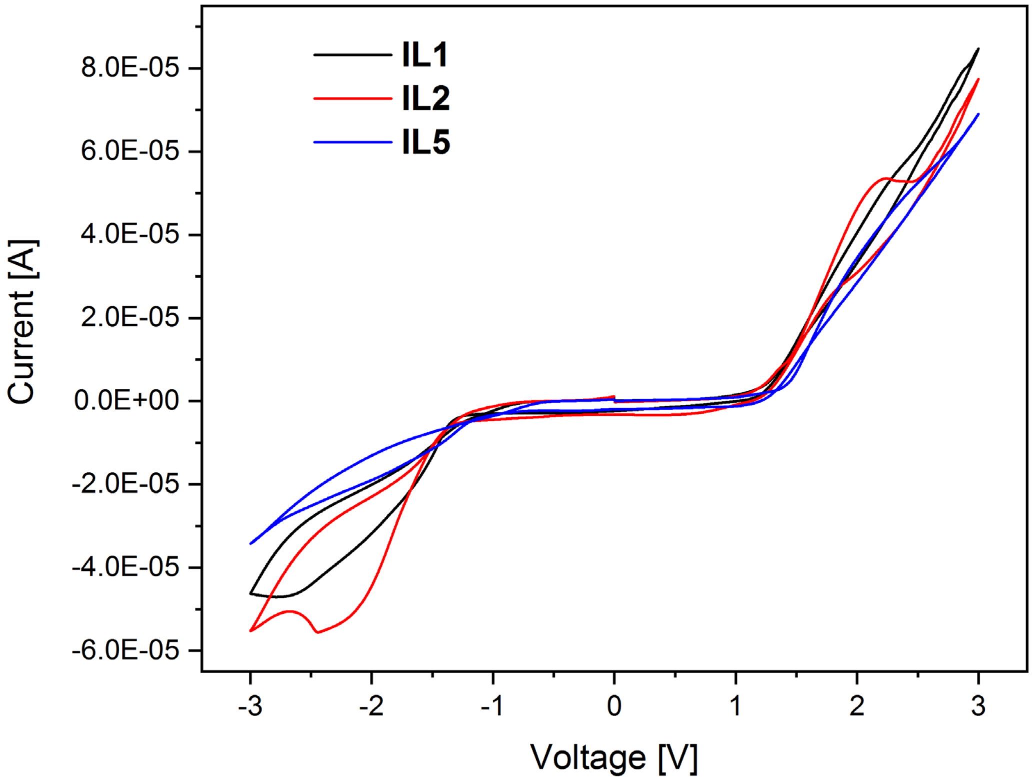

Figure S7. Cyclic volltamograms of **MILs 1, 2** and **5** in acetonitrile without the use of ferrocene as a reference.

5. Special Case: $(BuPy)_2[Cu_{0.25}Co_{0.75}Br_4]$

a. ICP-OES

Table S 4. *Metal contents in the ILs determined via ICP-OES measurements.*

Compound (theoretical composition from synthesis)	Cu [atom%]	Co [atom%]
$(C_4Py)_2[Cu_{0.25}Co_{0.75}Cl_4]$	27.68 ± 0.35	72.32 ± 0.35
$(C_4Py)_2[Cu_{0.25}Co_{0.75}Br_4]$	24.42 ± 0.06	75.58 ± 0.06

b. Thermal Properties

i. Differential Scanning Calorimetry

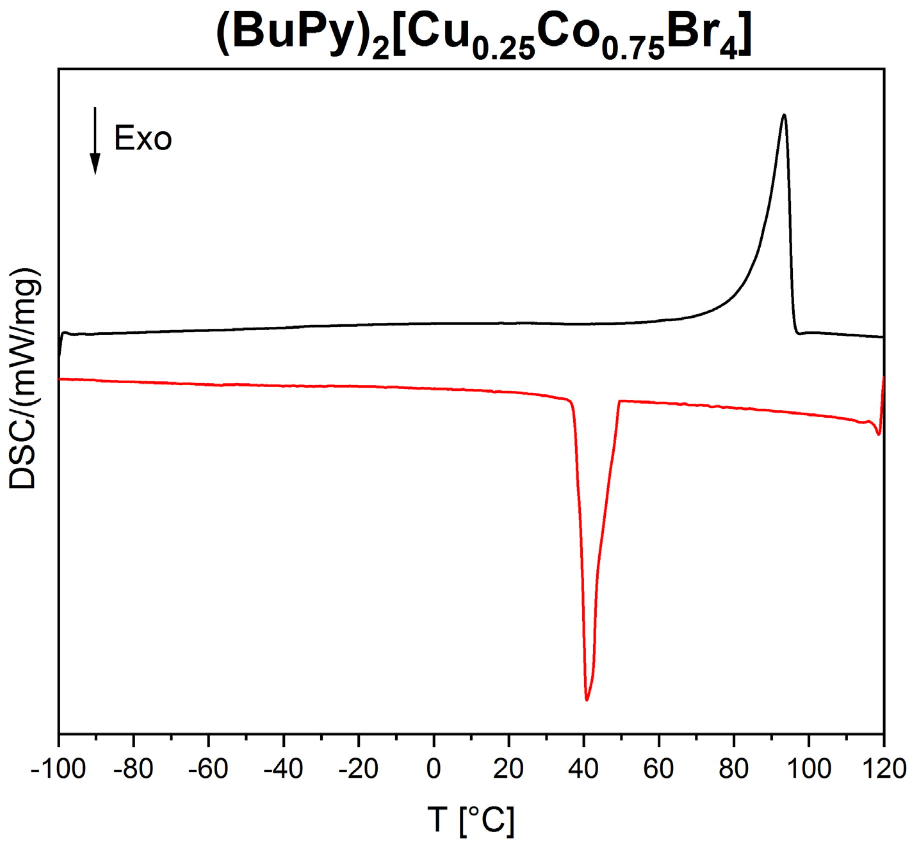

Figure S8. Third heating (black) and cooling (red) cycle of $(BuPy)_2[Cu_{0.25}Co_{0.75}Br_4]$ in DSC.

ii. Thermal Gravimetric Analysis

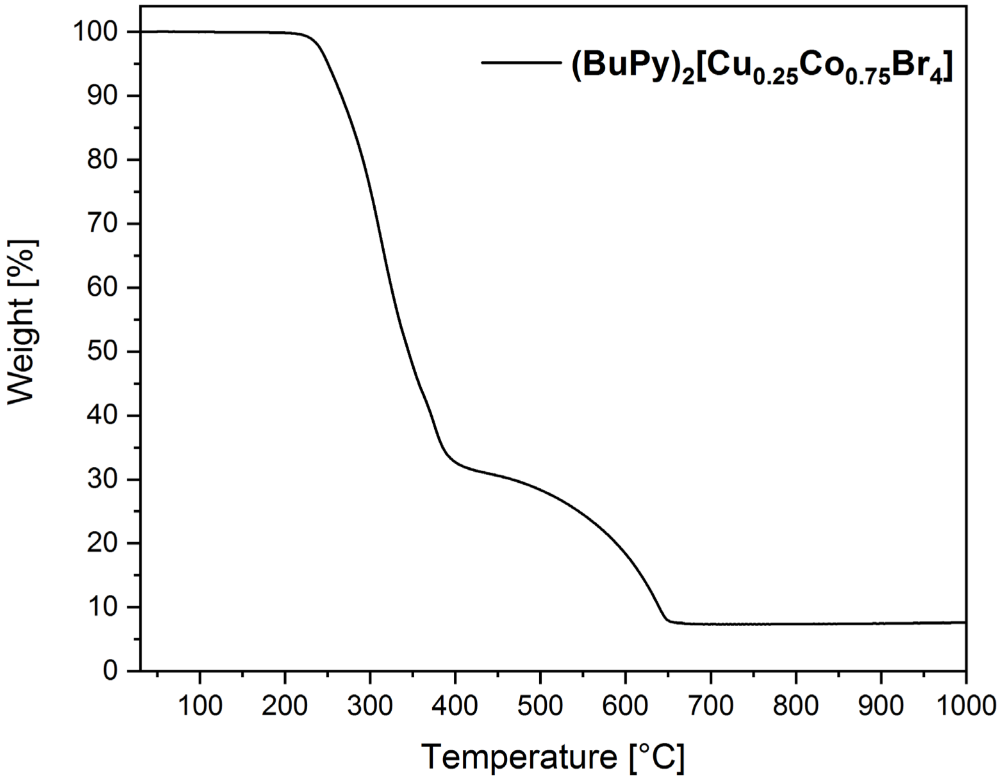

Figure S9. TGA data of $(BuPy)_2[Cu_{0.25}Co_{0.75}Br_4]$.

c. Conductivity

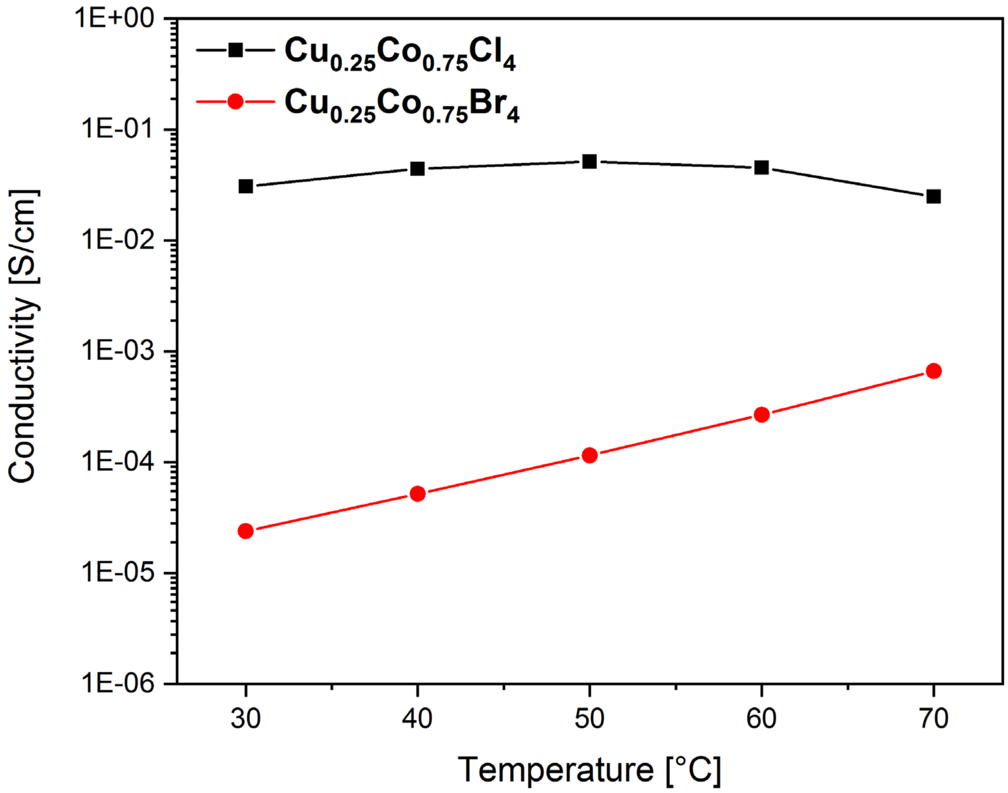

Figure S10. Conductivities of $(BuPy)_2[Cu_{0.25}Co_{0.75}Br_4]$ between 30 and 70°C in comparison to $(BuPy)_2[Cu_{0.25}Co_{0.75}Cl_4]$.

Table S 5. *Conductivity data of $(BuPy)_2[Cu_{0.25}Co_{0.75}Br_4]$ between 30 and 70°C in comparison to $(BuPy)_2[Cu_{0.25}Co_{0.75}Cl_4]$. Note: [a] Temperature, [b] Resistance of bulk phase, [c] Resistance error, [d] Conductivity.*

MIL	*T* [°C][a]	*R* [Ω][b]	*ΔR* [Ω][c]	*σ* [S cm^{-1}][d]
**$(BuPy)_2[Cu_{0.25}Co_{0.75}Cl_4]$**	30	3.52E+02	± 0.58	3.06E-02
	40	2.42E+02	± 0.15	4.44E-02
	50	2.10E+02	± 0.27	5.13E-02
	60	2.38E+02	± 0.13	4.53E-02
	70	4.33E+02	± 2.17	2.49E-02
**$(BuPy)_2[Cu_{0.25}Co_{0.75}Br_4]$**	30	3,63E+05	± 1,70E+03	2,39E-05
	40	1,68E+05	± 7,86E+02	5,19E-05
	50	7,56E+04	± 3,24E+02	1,15E-04
	60	3,25E+04	± 7,96E+01	2,68E-04
	70	1,31E+04	± 1,25E+01	6,65E-04

A preliminary study of the MIL $(BuPy)_2[Cu_{0.25}Co_{0.75}Br_4]$ (with a composition where there is a 0.25:0.75 metal:metal ratio rather than the 50:50 metal:metal ratio described in the main article) in comparison with the previously published $(BuPy)_2[Cu_{0.25}Co_{0.75}Cl_4]$ supports our current study. The melting point of the chloride MIL is higher than the bromide one, which is probably the result of stronger hydrogen bonds, which lead to a chain-like structure compared to a dimer-like structure. The conductivities of the chloride MIL are also higher. This is mainly due to two effects: 1) The formation of chain-structures in the crystal structure increases the ion movement due to its ion-guiding effect and 2) the presence of chloride in the MIL leads to a very reactive and corrosive behavior. As a result of the high redox potential of Cl^- and the high electronegativity coupled with its smaller size, the chloride MILs can attack the gold coating and afterwards the underlying nickel core of the electrodes during the impedance measurement. This damages the electrodes and the MIL, while also leading to the formation of side products, such as CuCl, that increase the conductivity.

Consequently, the aforementioned tradeoff between conductivity vs. stability is confirmed through this preliminary study. This shows that although the chloride analogue shows higher conductivities at first, the $(BuPy)_2[Cu_{0.25}Co_{0.75}Br_4]$ MIL is clearly more suitable for electrochemical applications as it is not destroyed during the measurement.
